# Inhibition of NF-κB Activation In Vivo Impairs Establishment of Gammaherpesvirus Latency

**DOI:** 10.1371/journal.ppat.0030011

**Published:** 2007-01-26

**Authors:** Laurie T Krug, Janice M Moser, Shelley M Dickerson, Samuel H Speck

**Affiliations:** 1 Department of Microbiology and Immunology, Emory University School of Medicine, Atlanta, Georgia, United States of America; 2 The Emory Vaccine Center, Emory University School of Medicine, Atlanta, Georgia, United States of America; University of Wisconsin-Madison, United States of America

## Abstract

A critical determinant in chronic gammaherpesvirus infections is the ability of these viruses to establish latency in a lymphocyte reservoir. The nuclear factor (NF)-κB family of transcription factors represent key players in B-cell biology and are targeted by gammaherpesviruses to promote host cell survival, proliferation, and transformation. However, the role of NF-κB signaling in the establishment of latency in vivo has not been addressed. Here we report the generation and in vivo characterization of a recombinant murine gammaherpesvirus 68 (γHV68) that expresses a constitutively active form of the NF-κB inhibitor, IκBαM. Inhibition of NF-κB signaling upon infection with γHV68-IκBαM did not affect lytic replication in cell culture or in the lung following intranasal inoculation. However, there was a substantial decrease in the frequency of latently infected lymphocytes in the lung (90% reduction) and spleens (97% reduction) 16 d post intranasal inoculation. Importantly, the defect in establishment of latency in lung B cells could not be overcome by increasing the dose of virus 100-fold. The observed decrease in establishment of viral latency correlated with a loss of activated, CD69^hi^ B cells in both the lungs and spleen at day 16 postinfection, which was not apparent by 6 wk postinfection. Constitutive expression of Bcl-2 in B cells did not rescue the defect in the establishment of latency observed with γHV68-IκBαM, indicating that NF-κB–mediated functions apart from Bcl-2–mediated B-cell survival are critical for the efficient establishment of gammaherpesvirus latency in vivo. In contrast to the results obtained following intranasal inoculation, infection of mice with γHV68-IκBαM by the intraperitoneal route had only a modest impact on splenic latency, suggesting that route of inoculation may alter requirements for establishment of virus latency in B cells. Finally, analyses of the pathogenesis of γHV68-IκBαM provides evidence that NF-κB signaling plays an important role during multiple stages of γHV68 infection in vivo and, as such, represents a key host regulatory pathway that is likely manipulated by the virus to establish latency in B cells.

## Author Summary

A central aspect of chronic infection of a host by herpesviruses is the ability of these viruses to establish a quiescent infection (latent infection) in some cell type(s) in which there is only intermittent production of progeny virus (virus reactivation). The establishment of a latent infection in the antibody producing cells of the host immune system (B lymphocytes) is critical for life-long persistence of gammaherpesviruses, as well as the development of virus-associated lymphoproliferative diseases (e.g., B-cell lymphomas). Nuclear factor (NF)-κB transcription factors are a family of cellular proteins that play an important role regulating gene expression in B cells, and it has been shown that gammaherpesviruses have evolved multiple strategies for manipulating NF-κB activity. However, to date there has been no reported examination of the role of NF-κB in the establishment of chronic gammaherpesvirus infection in vivo. Murine gammaherpesvirus 68 (γHV68) infects rodents and shares genetic and biologic properties with the human gammaherpesviruses, Epstein-Barr virus and Kaposi sarcoma–associated herpesvirus. To selectively block the function of NF-κB in infected cells, we engineered a transgenic virus that expresses a repressor of NF-κB activation (IκBαM). Notably, this recombinant virus was defective in the establishment of latency in B cells in the lungs and spleen following intranasal inoculation. We also observed that the decrease in B-cell infection could not be rescued by forced expression of the cellular Bcl-2 protein, which is normally upregulated by NF-κB and serves to protect B cells from some forms of cell death. Thus, we conclude that NF-κB is an important host factor for the successful establishment of a chronic infection by gammaherpesviruses, and likely requires functions of NF-κB apart from its role in B-cell survival.

## Introduction

Murine gammaherpesvirus 68 (γHV68) shares many genetic and biologic properties with its human counterparts, Epstein-Barr virus (EBV) and Kaposi sarcoma–associated herpesvirus (KSHV or HHV-8). For example, it has been shown for both EBV and γHV68 that long-term latency is maintained in memory B cells [1–3]. Identifying the host-dependent requirements for gaining access to the latency reservoir is an important step toward understanding how the virus modulates the host to establish a chronic infection. Such virus–host interactions may lead to dysregulation of normal cellular controls, increasing the risk for the development of lymphomas and other tumors etiologically associated with gammaherpesvirus infections [4,5].

Nuclear factor (NF)-κB transcription factors are key regulatory molecules of genes involved in innate and adaptive immunity. The absence of particular NF-κB subunits, or upstream regulatory molecules, can result in defects in B-cell development and functions such as activation-induced proliferation (reviewed in [6–8]). The maturation of B cells and survival in the periphery involve NF-κB–mediated upregulation of antiapoptotic *bcl-2, bcl-xL,* and *bfl-1/A1* genes [7,9]. The proliferative response of B cells to stimulation requires NF-κB–mediated upregulation of *c-Myc, cyclins,* and *CDK4* [7,10,11].

The NF-κB family of transcription factors is comprised of the subunits p65 (RelA), cRel, RelB, p50 (NF-κB1), and p52 (NF-κB2) that form dimers to mediate sequence-specific regulation of gene expression upon activation. Dimers of NF-κB subunits are retained in the cytoplasm by inhibitory IκB molecules. Cellular activation leads to proteosomal-dependent degradation of IκB molecules and translocation of NF-κB dimers to the nucleus. The engagement of cell surface receptors such as the B-cell receptor, receptors for inflammatory cytokines (e.g., tumor necrosis factor [TNF] α), and Toll-like receptors lead to the release of p50:cRel and p50:relA dimers through the classic pathway. The phosphorylation and subsequent degradation of the inhibitor of NF-κB, IκBα, are mediated by the IκB kinase (IKK) complex (IKK1, IKK2, and NEMO). In contrast, engagement of receptors such as the lymphotoxin β receptor and the B-cell–activating factor of the TNF family (BAFF) leads to the release of p52:RelB dimers via the alternative IKK1/NIK-dependent pathway (reviewed in [12]). Engagement of primary B cells with CD40L leads to the activation of NF-κB via both the classic and alternative pathways [11].

Given the critical role that NF-κB plays in B-cell biology, lymphotropic gammaherpesviruses have evolved multiple strategies to modulate NF-κB activity in infected cells. For example, the latent membrane protein 1 (LMP-1) encoded by EBV is expressed during the growth latency program and is essential for EBV immortalization of primary B cells [13,14]. LMP-1 drives NF-κB activation and is able to partially restore CD40-dependent functions in transgenic mice, indicating that it functions as a constitutively active CD40 receptor [15–17]. In the case of KSHV latency, the virus-encoded vFLIP has been shown to be essential for the proliferation and survival of primary effusion lymphoma cells, driving NF-κB activation via interactions with the TNF receptor–associated factors TRAF2 and TRAF3 [18–20]. Thus, it is likely that manipulation of NF-κB signaling is critical for the establishment and maintenance of gammaherpesvirus latency in vivo; however, to date this remains untested.

γHV68 infection of mice represents a tractable animal model in which both viral and host factors that modulate acute replication, latency, and reactivation can be addressed. Intranasal infection with γHV68 results in acute virus replication in the lungs, followed by B-cell–dependent spread of virus to the spleen, in turn followed by acute replication and establishment of latency in the spleen [21–25]. Subsequent to immune clearance of acute virus replication, multiple cell types, including lung epithelial cells, B cells, dendritic cells, and macrophages, are found to harbor the viral genome in the absence of preformed infectious virus [23,26–29]. Splenic B cells infected with γHV68 undergo proliferation and bear surface markers of cells that participate in germinal centers, sites of affinity maturation, and differentiation into long-lived memory B cells and antibody-producing plasma cells [2,27,30–32]. Notably, isotype-class–switched memory B cells are the predominant reservoir for latent γHV68 infection at late timepoints postinfection [2,3]. Thus, γHV68 pathogenesis has strong parallels with EBV infection in humans, where EBV latency is characterized by distinct latency programs involving differential expression of specific viral genes that modulate host signaling pathways to facilitate the proliferation of naïve cells into lymphoblasts, followed by differentiation of these lymphoblasts into memory B cells [13]. As with KSHV, γHV68 lacks clear homologs of the EBV latency-associated antigens, yet it has likely evolved a strategy for promoting the activation, proliferation, and differentiation of newly infected B cells into the memory B-cell reservoir.

NF-κB likely plays a critical role in multiple aspects of γHV68 pathogenesis. The role of one upstream activator of NF-κB, CD40, has been examined in the context of an intact immune response. Blackman et al. [3] infected CD40^+^/CD40^−^ mixed bone marrow chimeric mice and reported that CD40-deficient B cells latently infected with γHV68 rapidly waned compared to latency in CD40-sufficient B cells. This suggests that NF-κB activation upon CD40L stimulation plays a role in the maintenance of latency in B cells [3]. Finally, replication of γHV68 in cell culture is blocked by overexpression of p65, and it has been proposed that NF-κB activation may promote the establishment of latency by inhibiting lytic cycle initiation [33].

Here we sought to address the role of NF-κB signaling in vivo using virus-directed inhibition of NF-κB activation. This experimental approach avoids the global immune dysfunction present in NF-κB knockout mice, which would not allow the role of NF-κB activation in the establishment of viral latency in B cells to be readily distinguished from its role in the immune response and subsequent control of γHV68 infection. To this end, we report the generation and characterization of a recombinant γHV68 expressing a mutant form of the NF-κB inhibitor IκBα, IκBαM, that functions as a superrepressor of NF-κB activation. We find that while dispensable for in vitro replication, NF-κB activation is a critical host determinant for γHV68 latency in vivo.

## Results

### Targeted Disruption of NF-κB Signaling in γHV68-Infected Cells

**Figure 1:**
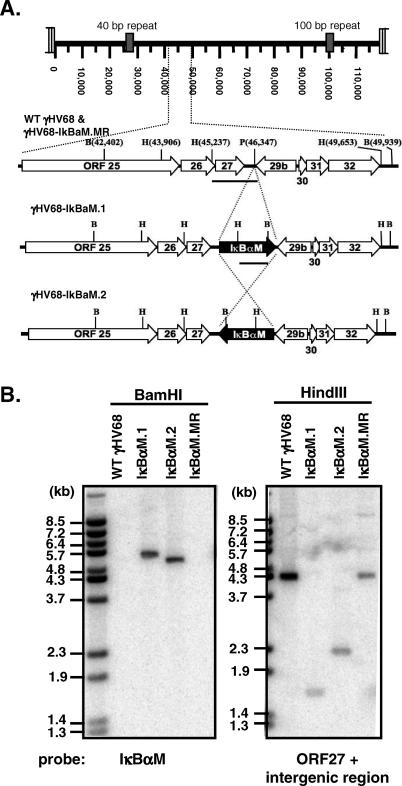
Construction and Verification of the γHV68-IκBαM Viruses (A) Genomic structure of WT γHV68, γHV68-IκBαM.1 and γHV68-IκBαM.2, and γHV68-IκBαM.MR in the ORF27-29b intergenic region. In γHV68-IκBαM.1 and .2 viruses, the 1.8-kb HCMV immediate-early promoter-driven IκBαM expression cassette was inserted at the PmlI site in either the rightward or leftward orientation, respectively. All genome coordinates are based on the γHV68 WUMS sequence. Bars below the genomic regions indicate regions used as probes in the Southern blot. B, BamHI; H, HindIII; P, PmlI. (B) Southern blot analysis of WT γHV68, γHV68-IκBαM.1 and γHV68-IκBαM.2, and γHV68-IκBαM.MR viral genomes. Viral DNA was purified from extracellular virions and subsequently digested with BamHI or HindIII, electrophoresed, blotted, and hybridized with a ^32^P-labeled probe as indicated. The fragment sizes of ^32^P-labeled BstEII-digested Lambda DNA are indicated.

To investigate the consequences of inhibiting the NF-κB signaling pathway during the infection of mice with γHV68, yet avoid global alterations in the immune response, we generated a recombinant γHV68 that would inhibit NF-κB signaling only in infected cells. To accomplish this, we used a mutant form of IκBα, IκBαM, that contains serine-to-alanine substitutions at amino acids 32 and 36, thus preventing phosphorylation, proteasomal degradation, and release of NF-κB dimers to the nucleus following appropriate upstream stimuli [34]. This “superrepressor” functions as a potent transdominant inhibitor of NF-κB activation in cell culture [35–37] and in vivo [38]. We inserted an IκBαM expression cassette, driven by the HCMV major immediate-early promoter (MIEP), into the intergenic region between ORFs 27 and 29b of γHV68 (Figure 1A). It has previously been shown that insertion of a HCMV MIEP-driven Cre recombinase expression cassette [32], insertion of a transposon [39] or the mutagenesis of ORF28 in this region [40] does not impair virus replication in vitro [39,40] or in vivo [40], and has no discernable impact on the establishment of, or reactivation from, splenic latency in C57BL/6 mice [32,40].

The IκBαM.1 and IκBαM.2 recombinant viruses were generated as described in Materials and Methods through allelic exchange in Escherichia coli utilizing a γHV68-BAC [41] followed by removal of BAC sequences through Cre-mediated recombination in Vero-Cre cells. We generated two independent recombinant γHV68 viruses, IκBαM.1 and IκBαM.2, which contain the IκBαM expression cassette in either the rightward or leftward orientation within the viral genome, respectively (Figure 1A). In addition, two independent marker rescue viruses were generated through allelic exchange with IκBαM.1, one of which (IκBαM.MR) was fully characterized here. As shown in Figure 1B, Southern analysis of BamHI-digested viral DNA isolated from extracellular virions, followed by hybridization with a [^32^P]-labeled probe specific for IκBαM, detected the IκBαM insert only in the IκBαM.1 and IκBαM.2 recombinant viruses (revealing the expected product sizes of 5,477 bp and 5,127 bp, respectively). A probe containing a portion of ORF27 and the ORF27-29b intergenic region identified the expected 4,416-bp fragment in HindIII-digested wild-type (WT) and IκBαM.MR viral DNAs. As expected, the introduction of a new HindIII site within the IκBαM insert resulted in a reduction in the sizes of the hybridizing fragments for the recombinant viruses harboring the IκBαM expression cassette in the rightward (IκBαM.1; 1,682 bp) or leftward (IκBαM.2; 2,321 bp) orientation (Figure 1B).

**Figure 2:**
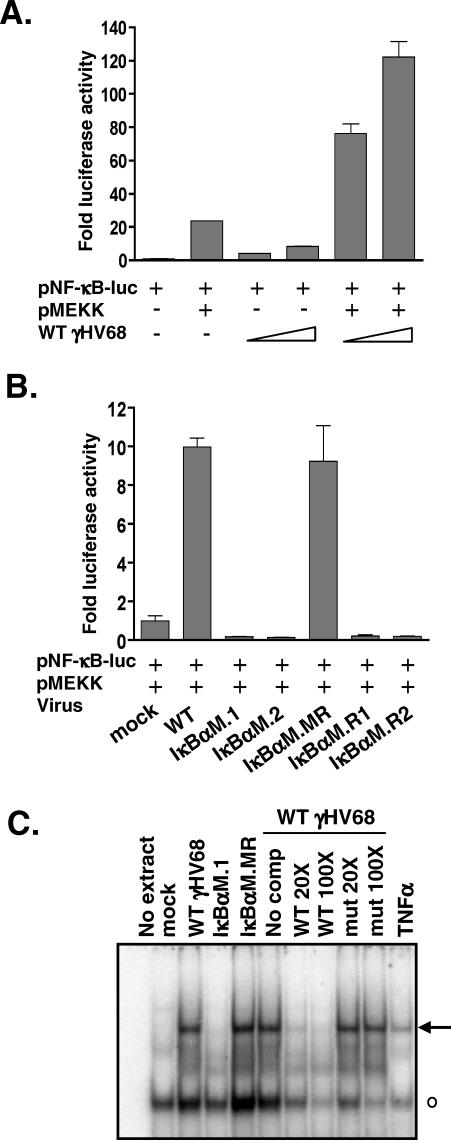
γHV68-IκBαM Inhibits NF-κB Signaling in Fibroblasts (A) NIH 3T12 fibroblasts were infected with WT virus at an MOI of 1 or 10 for 6 h and then transfected with a NF-κB–responsive luciferase reporter construct and, where indicated, a plasmid expressing MEKK1. Data are shown as the mean fold activation of the NF-κB promoter over mock infected samples ±SD of triplicate wells. (B) NIH 3T12 cells were infected with the indicated viruses at an MOI of 10 for 6 h and then transfected with the NF-κB luciferase reporter construct and a plasmid expressing MEKK1. At 24 h posttransfection, wells were harvested and luciferase activity was quantitated. Data are shown as the mean fold activation of the NF-κB promoter over mock infected, MEKK1-transfected samples ±SD of triplicate wells and are representative of multiple independent experiments. IκBαM.R1 and IκBαM.R2 were viruses recovered from reactivating splenocytes harvested from mice infected with either the IκBαM.1 or IκBαM.2 recombinant virus, respectively. (C) Electrophoretic mobility shift analysis of NF-κB binding using nuclear extracts prepared from NIH 3T12 cells infected with the indicated viruses at an MOI of 3 for 48 h, as described in Materials and Methods. Also shown, as a positive control for NF-κB induction, is treatment of uninfected NIH 3T12 cells with 25 ng/ml TNFα for 1 h prior to harvest. Specific (arrow) and nonspecific (open circle) complexes are indicated.

The ability of the IκBαM-expressing viruses to inhibit NF-κB activation in the context of virus infection was examined using an NF-κB–responsive reporter construct in infected cells. NIH 3T12 fibroblasts were infected with WT γHV68 at a multiplicity of infection (MOI) of 1 or 10 for 6 h followed by transfection with an NF-κB–dependent luciferase construct, in the presence or absence of an expression construct for MEKK1. At 24 hours postinfection (hpi), cells were harvested and luciferase activity was quantitated. Notably, infection with WT γHV68 activated the NF-κB–dependent reporter construct in a dose-dependent manner to levels nearly 8-fold over uninfected samples (Figure 2A). This activation synergized with MEKK1 activation to further enhance NF-κB activation of the reporter construct over 100-fold compared to mock infected samples (Figure 2A). In an independent experiment, the synergistic induction of the NF-κB–responsive reporter construct by MEKK1 and γHV68 infection was used to examine the recombinant IκBαM-expressing viruses. Consistent with the results shown in Figure 2A, infection of WT or IκBαM.MR virus enhanced MEKK1 activation of the NF-κB–responsive reporter construct approximately 10-fold above that observed in mock infected cells (Figure 2B). In contrast, both IκBαM-expressing viruses strongly inhibited MEKK1 activation of the NF-κB–responsive reporter construct (Figure 2B). Analysis of NF-κB activity in cells infected with a recombinant γHV68 harboring a CMV MIEP-driven CRE-recombinase expression cassette in the intragenic region between ORFs 27 and 29b revealed no inhibition of NF-κB activation, arguing against interference with NF-κB activation due to insertion of the CMV MIEP-driven expression cassette in this region of the viral genome (unpublished data). Taken together, these data directly demonstrate effective inhibition of NF-κB activation by the recombinant viruses expressing IκBαM, in response to both virus infection and MEKK1-driven activation.

To further examine activation of NF-κB upon γHV68 infection of murine fibroblasts, nuclear extracts were prepared from mock infected NIH 3T12 cells, as well as from NIH 3T12 cells infected with either WT γHV68, IκBαM.1, or IκBαM.MR. Electrophoretic mobility shift analysis of nuclear extracts incubated with a consensus NF-κB binding site revealed the induction of NF-κB binding activity in WT and IκBαM.MR virus–infected cells but not in the IκBαM.1-infected cells (Figure 2C). Furthermore, the induced NF-κB complex could be competed by an unlabeled double-stranded oligonucleotide probe containing a consensus NF-κB binding site but not by a competitor containing a mutated NF-κB binding site (Figure 2C). Electrophoretic mobility supershift analyses of the induced NF-κB complex, using antibodies targeted against specific NF-κB family members, demonstrated that the induced complex contains p65 but does not appear to contain either c-rel or p50 (unpublished data).

### NF-κB Activation Is Dispensable for Virus Replication in Tissue Culture or In Vivo in the Lung

**Figure 3:**
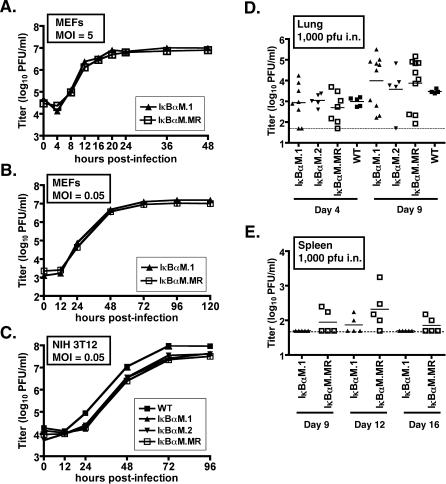
Inhibition of NF-κB Signaling Does Not Impair Lytic Replication In Vitro or Acute Replication in the Lungs (A) Single-step growth curve in MEFs, with an MOI of 5.0 PFU per cell with the indicated virus. Samples were harvested at the indicated time points and titers were determined on NIH 3T12 fibroblasts as described in Materials and Methods. (B) Multistep growth curve in MEFs, with an MOI of 0.05 PFU per cell with the indicated viruses. (C) Multistep growth curve in NIH 3T12 cells, with an MOI of 0.05 PFU per cell with the indicated viruses. Data are representative of at least two independent experiments. (D and E) C57Bl6 mice were infected with 1,000 PFU by the intranasal route of inoculation with the indicated viruses. On the indicated days postinfection, lungs (D) and spleens (E) were harvested, disrupted, and titered on NIH 3T12 cells. The data were compiled from one or two experiments with three to five mice analyzed per experiment. Data are shown as log10 titer, and the bar indicates the geometric mean titer. The dashed line indicates the limit of detection of this assay as log101.7 or 50 PFU/ml of sample homogenate.

The ability to easily generate high-titer viral stocks from the initial transfection of γHV68-IκBαM BAC DNA into Vero-Cre cells indicated that insertion of the IκBαM expression cassette into the viral genome does not appreciably impair viral replication in vitro. To quantitatively assess the role of NF-κB signaling during lytic replication, we compared replication of IκBαM.1 and IκBαM.MR in primary mouse embryonic fibroblasts (MEFs). MEFs were infected for single-step (MOI = 5.0) and multistep (MOI = 0.05) growth assays, followed by analysis of virus growth at various times postinfection by plaque assay on NIH 3T12 cells. Notably, the kinetics of virus replication in the single-step (Figure 3A) and multistep (Figure 3B) growth curves were nearly indistinguishable between IκBαM.1 and IκBαM.MR viruses. The replication of IκBαM.1, and IκBαM.2, IκBαM.MR, and WT γHV68 in NIH 3T12s was also compared in a multistep growth analysis (Figure 3C). Consistent with the analyses shown in Figure 3A, all viruses replicated with similar kinetics and to comparable virus titers across the 96-h time course (Figure 3C). These data demonstrate that the insertion of the IκBαM expression construct into the viral genome (in the intragenic region between ORFs 27 and 29b), and the resulting inhibition of NF-κB signaling by IκBαM expression, did not negatively impact γHV68 replication in vitro.

Upon intranasal infection of mice, γHV68 undergoes a lytic replicative expansion in the lung epithelium prior to trafficking and seeding of other target organs and tissues, such as the spleen. The ability of the recombinant viruses expressing IκBαM to replicate in the lungs of C57BL/6 (BL6) mice during this acute phase was examined (Figure 3D). At days 4 and 9, following intranasal inoculation with 1,000 PFU of virus, lungs were harvested, disrupted and virus titers determined. Acute viral titers in mice infected with the IκBαM.1 and IκBαM.2 recombinant viruses were comparable to that observed in mice infected with either WT virus or IκBαM.MR (Figure 3D). Thus, we conclude that NF-κB activation is dispensable for productive replication in the lungs during the acute phase of virus infection. Interestingly, productive replication of the IκBαM-expressing virus following low-dose intranasal inoculation was slightly impaired when compared to IκBαM.MR, although replication of both the IκBαM.MR- and the IκBαM-expressing virus in the spleen was very low (or not detectable) at all times postinfection (Figure 3E). We have previously reported that low-dose intranasal inoculation (40 PFU) leads to delayed seeding and clearance of acute virus replication in the lungs and the spleen compared to high-dose inoculation (4 × 10^5^ PFU) [25]. However, IκBαM.1 was observed to replicate with normal replication kinetics in the lung (Figure 3D), and the low level of lytic replication of IκBαM.1 in the spleen provided no evidence for delayed clearance of acute replication upon infection with IκBαM.1. As discussed below, a defect in acute IκBαM.1 replication in the spleen was also noted at day 9 post intraperitoneal inoculation and indicates a role for NF-κB during lytic expansion in the spleen.

### Inhibition of NF-κB Signaling Impairs Establishment of Splenic Latency

**Figure 4:**
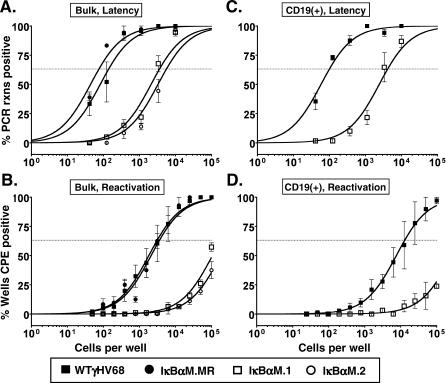
Splenic Latency Is Markedly Reduced in Mice Infected with 1,000 PFU of γHV68-IκBαM by the Intranasal Route of Inoculation Bulk splenocytes or flow cytometry-sorted CD19^+^ splenic cell populations were harvested from infected C57BL/6 mice at 15 or 16 dpi and analyzed by limiting-dilution viral genome PCR (A and C) and limiting-dilution ex vivo reactivation assays (B and D) as described in Materials and Methods. (A) Frequency of unsorted, bulk splenocytes harboring viral genomes. (B) Frequency of unsorted, bulk splenocytes reactivating virus. Significant levels of preformed virus were not detected. (C) Frequency of sorted CD19^+^ B cells harboring viral genome. Using a PE-conjugated antibody to the pan–B-cell marker CD19 (CD19-PE), splenic B cells were separated into B-cell (CD19^+^) and non–B-cell (CD19^−^) populations isolated by FACS. Postsort FACS analysis indicated that the mean purities for CD19^+^ cells were 97.7 ± 1.1% for WT and 96.7 ± 3.4% for IκBαM.1. (D) Frequency of sorted CD19^+^ B cells reactivating virus. For both limiting-dilution assays, curve fit lines were derived from nonlinear regression analysis, and symbols represent the mean percentage of wells positive for virus (viral DNA or cytopathic effect) ±SEM. The dashed line represents 63.2%, from which the frequency of viral genome–positive cells or the frequency of cells reactivating virus was calculated based on Poisson distribution. The data shown represent three to five independent experiments with spleen cells pooled from five mice per experimental group. Where examined, the frequency of splenocytes and CD19^+^ B cells that harbored viral genome in mice infected with IκBαM.1 or IκBαM.2 was significantly different (*p* < 0.02) from that of mice infected with WT or IκBαM.MR control viruses.

Since impairment of NF-κB signaling had no effect on virus replication in the lung, yet slightly impaired acute replication in the spleen, we next examined the role of NF-κB signaling in the establishment of latency, as well as reactivation from latency, in bulk splenocytes and specific splenocyte subpopulations at early times postinfection. Mice were infected via intranasal inoculation with 1,000 PFU of IκBαM.1, IκBαM.2, IκBαM.MR, or WT γHV68. At 15 to 16 days postinfection (dpi), splenocytes were isolated and the frequency of viral genome–positive cells was determined by limiting-dilution PCR analysis (Figure 4A). Unsorted, bulk splenocytes were found to harbor viral genome at a frequency of one in 3,646 and one in 5,570 cells in mice infected with either the IκBαM.1 and IκBαM.2 recombinant virus, respectively. This was a significantly lower frequency than that observed in mice infected with either WT γHV68 (one in 138 cells) or IκBαM.MR virus (one in 75 cells) (Figure 4A, summarized in Table 1). This approximately 50-fold reduction represented a greater than 96% decrease in the frequency of latently infected cells in mice infected with the IκBαM-expressing recombinant viruses compared to WT and marker rescue control viruses, indicating that inhibition of NF-κB signaling has a substantial impact on the ability of γHV68 to establish latency in splenocytes following intranasal infection.

**Table 1:**
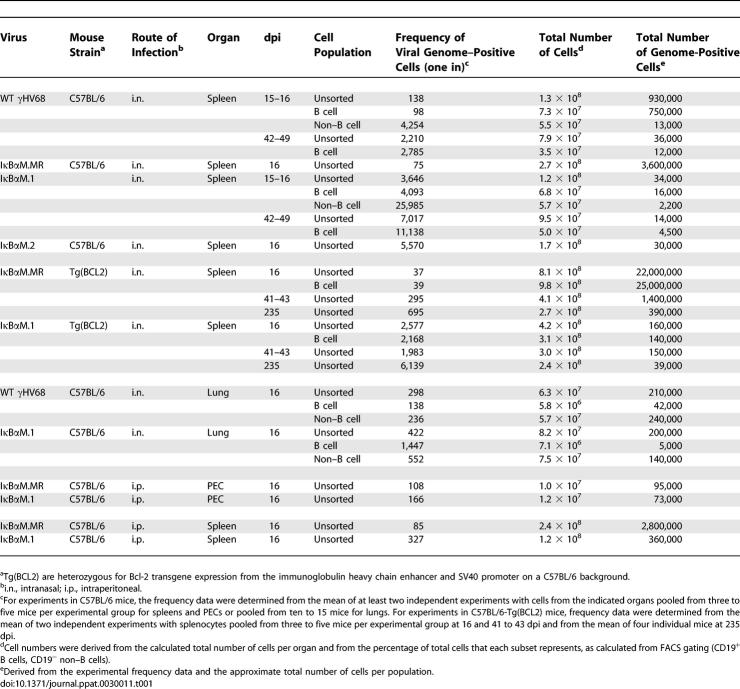
Frequencies of Cell Populations Harboring Viral Genomes

To determine the ability of splenocytes harboring the IκBαM virus to reactivate virus, we quantitated ex vivo reactivation of intact cells by limiting-dilution analysis on MEFs as described in Materials and Methods. Splenocytes from WT and IκBαM.MR-infected mice reactivated at a frequency of one in 3,102 and one in 3,563, respectively (Figure 4B). The frequency of reactivation of bulk splenocytes from mice infected with IκBαM.1 and IκBαM.2 was significantly lower than control virus reactivation and required a mathematical extrapolation of the best-fit curve, leading to estimated frequencies of one in 180,936 and one in 300,788 for IκBαM.1 and IκBαM.2, respectively (Figure 4B). The amount of preformed infectious virus detected by plating mechanically disrupted cells in parallel was negligible for all samples. The reactivation frequencies of the IκBαM recombinant viruses are approximately 50-fold lower than the frequency of viral genome–positive cells, in close agreement with the decrease in the frequency of viral genome–positive splenocytes. Thus, the efficiency of virus reactivation is similar for cells infected with the IκBαM recombinant viruses and the control viruses.

It is unclear whether the residual levels of latency observed in mice infected with the IκBαM expressing viruses represent the infection of a population of cells that do not require NF-κB activation for establishment of γHV68 latency or whether they arise from latently infected cells in which the levels of expression of IκBαM were not sufficient to block NF-κB activation. Unfortunately, it is not possible at this time to distinguish between these alternatives. We also considered the possibility that infection with the IκBαM-expressing viruses could give rise in vivo to a revertant virus that has lost the ability to express a functional IκBαM. To address this possibility, independent isolates of virus (IκBαM.R1 and IκBαM.R2) were recovered from ex vivo reactivation of splenocytes harvested from IκBαM-infected mice and analyzed for the presence of the IκBαM expression cassette. Southern blot analyses revealed that these viral genomes had retained the IκBαM expression cassette (unpublished data). More important, the viruses recovered from reactivated splenocytes also retained their ability to inhibit NF-κB activation in NIH 3T12 fibroblasts (Figure 2B). Therefore, the small population of cells that is viral genome–positive appears to harbor virus capable of expressing IκBαM.

To examine the role of specific splenocyte cell populations, bulk splenocytes were separated by fluorescence-activated cell sorting (FACS) into B-cell and non–B-cell populations utilizing CD19 as a marker for B cells. This resulted in cell population of greater than 93% purity for each subset (unpublished data). As shown in Figure 4C and summarized in Table 1, the CD19^+^ B-cell population was found to harbor viral genome–positive cells at a similar frequency as the bulk splenocyte population following WT γHV68 infection. This is consistent with previous reports that B cells comprise the major reservoir of γHV68 latency in the spleen [2,27,42,43]. As observed with bulk splenocytes, CD19^+^ B cells from mice infected with IκBαM.1 had a drastic reduction in the frequency of latently infected cells (one in 4,093) compared to those from mice infected with WT (one in 98). This 40-fold difference represents a 98% reduction in the establishment of latency upon NF-κB inhibition, which was further substantiated by an estimated 98% reduction in the frequency of CD19^+^ cells reactivating virus in IκBαM.1-infected mice compared to mice infected with WT (Figure 4D). The frequency of latently infected CD19^−^ non–B cells was also diminished in mice infected with IκBαM.1 by 6-fold (84% reduction) compared to those infected with WT virus (Table 1). At this point, it is unclear whether the diminished levels of latency in the non–B-cell population reflect a dependence on B-cell latency for establishment of latency in the non–B-cell reservoirs or whether there is a role for NF-κB activation in the establishment of latency in the non–B-cell reservoirs. Regardless, these data indicate that NF-κB signaling is critical for the efficient establishment of latency in splenic B cells following intranasal inoculation.

### Virus Replication Does Not Contribute to Changes in Viral Load between Early and Intermediate Times Postinfection

**Figure 5:**
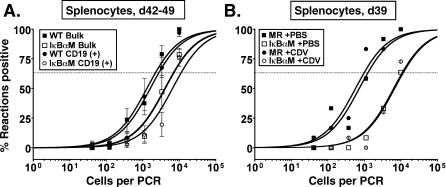
γHV68-IκBαM–Infected Mice Have Decreased Splenic Latency at Later Timepoints after Intranasal Infection that Is Not Altered by Inhibiting Lytic Replication Bulk splenocytes or FACS-sorted CD19^+^ splenic cell populations were harvested from infected C57BL/6 mice and analyzed by limiting-dilution viral genome PCR. (A) Frequency of unsorted, bulk and CD19^+^ B cells from mice infected with 1,000 PFU of WT γHV68 or IκBαM.1 harboring viral genomes at 42 and 49 dpi. Postsort FACS analysis indicated that the mean purities for CD19^+^ cells were 97.8 ± 1.3% for WT and 98.5 ± 1.5% for IκBαM.1. Data represent three independent experiments with spleen cells pooled from five mice per experimental group. The frequency of splenocytes and CD19^+^ B cells that harbored viral genome in mice infected with IκBαM was significantly different from that of mice infected with WT virus in an unpaired *t-*test as follows: *p* = 0.0146 (unsorted splenocytes) and *p* = 0.0198 (CD19^+^). (B) The effect of treatment with the antiviral drug cidofovir between 16 and 39 dpi on the frequency of viral genome–positive cells in mice infected with IκBαM.MR or IκBαM.1. Mice were injected subcutaneously with 100 μl of PBS or with 25 mg/kg cidofovir (CDV) in 100 μl of PBS every 3 d between 16 and 39 dpi. Mice were weighed weekly, and dosage was adjusted. Data represent one (PBS) or two (CDV) independent experiments of three to six mice per experimental group. The frequency of splenocytes that harbored viral genome in the mice infected with IκBαM.1 was significantly different (*p* = 0.0351) from that of mice infected with IκBαM.MR. Symbols represent the mean percentage of wells positive for virus (viral DNA) ±SEM.

Latency in WT-infected mice is characterized by a peak in viral genome–positive splenocytes at 16 dpi, a rapid contraction of latently infected splenocytes that is apparent by 6 wk postinfection, followed by a more gradual rate of decline throughout later time points as the virus reaches a steady-state level in memory B cells [2]. However, some γHV68 mutants that exhibit significant defects in the establishment of splenic latency (measured at 16 dpi) fail to contract at the same rate as WT virus and, as such, the frequency of latently infected splenocytes often approaches the level observed with WT virus infection at later times postinfection [44,45]. We therefore examined the frequency of latently infected splenocytes at 42 to 49 dpi (Figure 5A). In mice infected with IκBαM.MR, the frequency of viral genome–positive, unsorted splenocytes dropped 16-fold at 42 to 49 dpi from that observed at day 16, similar to previous reports with WT γHV68 [2,25]. In contrast, the frequency of unsorted splenocytes latently infected with IκBαM.1 decreased only 2-fold. Therefore, the frequency of viral genome–positive unsorted splenocytes infected with IκBαM.1 (one in 7,017) were only 3-fold lower than WT γHV68 (one in 2,210) by 42 to 49 dpi (*p* = 0.0146) (Figure 5A). As B cells represent the major long-term reservoir of viral latency in the spleen, the frequencies of genome-positive CD19^+^ B cells for IκBαM.1 (one in 11,138) and WT virus (one in 2,785) were very similar to the frequency of bulk splenocytes, where only a 4-fold difference between the frequencies of IκBαM and WT virus was apparent by 42 to 49 dpi (*p* = 0.0198) (Figure 5A).

We hypothesized that the dissimilar rates of contraction of latently infected splenocytes between IκBαM.1 and WT virus could involve differences in cell turnover due to lytic events and/or the reseeding of latency in the spleen by virus arising from in vivo reactivation (between day 16 and later time points) [46]. To assess whether virus reactivation and reseeding of latency might be contributing to the results observed at 42 to 49 dpi, we administered the antiviral drug cidofovir to mice using a drug dosage demonstrated in previous studies to effectively inhibit acute virus replication and seeding of the spleen by day 16 after intranasal infection of C57Bl/6 mice [47,48]. In this analysis, the frequency of viral genome–positive cells in control mice (no cidofovir) infected with the IκBαM.1 was 9-fold lower at 39 dpi compared to control mice infected with IκBαM.MR (Figure 5B). Surprisingly, the frequency of cells harboring viral genome in mice treated with cidofovir was nearly indistinguishable from that of the untreated mice infected with either IκBαM.1 or IκBαM.MR. This argues that the decline in latency of the IκBαM-expressing virus or the control IκBαM-MR virus between 16 and 39 dpi does not involve any significant contribution from virus reactivation and reseeding of splenic latency reservoirs and thus may reflect differences in trafficking of latently infected cells (see Discussion).

### Bcl2 Does Not Rescue the Latency Defect Observed with IκBαM-Expressing Viruses

**Figure 6:**
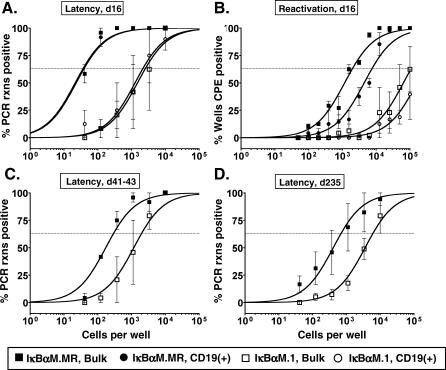
Transgenic Expression of Bcl-2 in B Cells Does Not Rescue the Defect in Splenic Latency Establishment by γHV68-IκBαM after Intranasal Infection Bulk unsorted splenocytes or magnetic bead–enriched CD19^+^ splenocytes from infected C57BL/6-Tg(BCL2) were analyzed by limiting-dilution viral genome PCR (A, C, and D) and limiting-dilution ex vivo reactivation assays (B) as described in Materials and Methods. (A) Frequency of unsorted, bulk splenocytes and CD19^+^ B cells from mice infected with 1,000 PFU of IκBαM.MR or IκBαM.1 harboring viral genomes at 16 dpi. Using a PE-conjugated antibody to the pan–B-cell marker CD19 (CD19-PE), splenic B cells were separated into B-cell (CD19^+^) and non–B-cell (CD19^−^) populations by magnetic activated cell sorting. Postsort FACS analysis indicated that the mean purities for CD19^+^ cells were 97.4 for WT, and 96.5 ± 1.5% for IκBαM.1. The frequency of splenocytes that harbored viral genome in mice infected with IκBαM was significantly different (*p* = 0.05) from that of mice infected with IκBαM.MR virus. (B) Frequency of unsorted, bulk splenocytes and CD19^+^ B cells reactivating virus from mice infected with 1,000 PFU of IκBαM.MR or IκBαM.1 at 16 dpi. The frequency of splenocytes and CD19^+^ B cells that harbored viral genome in mice infected with IκBαM was significantly different from that of mice infected with IκBαM.MR in a *t-*test as follows: *p* = 0.0460 (unsorted splenocytes) and *p* = 0.0286 (CD19^+^). (C) Frequency of unsorted, bulk splenocytes reactivating virus from mice infected with 1,000 PFU of IκBαM.MR or IκBαM.1 at 41 to 43 dpi. CD19^+^ B cells comprise greater than 67% of total splenocytes as determined by FACS analysis. (D) Frequency of unsorted, bulk splenocytes reactivating virus from mice infected with 1,000 PFU of IκBαM.MR or IκBαM.1 at 235 dpi. FACS analysis indicated that the unsorted splenocytes were composed of 66.3 ± 2.0% (IκBαM.MR) and 60.3 ± 7.7% (IκBαM.1) CD19^+^ B cells. Symbols represent the mean percentage of wells positive for virus (viral DNA or cytopathic effect) ±SEM. The data shown represent two independent experiments with cells pooled from three to five mice per experimental group (16 and 41 to 43 dpi) or one experiment with four individual mice at 235 dpi.

Bcl-2 is an antiapoptotic molecule whose expression is regulated by NF-κB. Notably, B-cell–specific expression of Bcl-2 in transgenic mice overcomes NF-κB–dependent requirements for mature B-cell development and survival in the periphery but does not restore B-cell proliferation upon mitogen activation [6]. To address the potential role of NF-κB as a factor critical for peripheral B-cell survival during γHV68 latency, we examined whether constitutive expression of Bcl-2 in B cells could rescue the establishment of latency in mice infected with IκBαM.1. On 16 dpi, there was an approximately 2-fold increase in the frequency of viral genome–positive unsorted splenocytes in Bcl-2 mice infected with IκBαM.MR compared to the frequency in infected C57BL/6 nontransgenic mice (Figure 6A and Table 1). However, there was no dramatic increase in the frequency of latency establishment in Bcl-2 mice infected with IκBαM.1 compared to infected C57BL/6 mice (Figure 6A and Table 1). Thus, the frequency of bulk, unsorted splenocytes from Bcl-2 mice infected with IκBαM.1 (one in 2,577) was 70-fold lower than the frequency of viral genome–positivity found in IκBαM.MR-infected mice (one in 37) (Figure 6A). This correlated with an absence of splenomegaly in IκBαM.1-infected mice as determined by spleen weight compared to uninfected mice (1.1-fold increase), while there was notable splenomegaly in the IκBαM.MR-infected mice (2.2-fold increase) (unpublished data).

Bcl-2 mice are characterized by a higher proportion of B cells in lymphoid organs, which represented 72% of the total splenocytes at 16 dpi in these studies. To further characterize latency in these transgenic mice, splenocytes harvested at 16 dpi were separated into CD19^+^ and CD19^−^ populations. As expected, the frequencies of CD19^+^ B cells harboring virus for both IκBαM.1 (one in 2,168) and IκBαM.MR (one in 39) (Figure 6A) were nearly identical to the frequencies observed with bulk splenocytes.

Since B cells from Bcl-2 transgenic mice are known to survive for a longer period of time in cell culture upon explant, we assessed whether latently infected splenocytes from Bcl2 mice would reactivate at a higher frequency than those recovered from C57BL/6 mice. Unsorted and CD19^+^ B cells were plated for ex vivo reactivation analyses at 16 dpi. As observed for splenocytes in the C57BL/6 mice, bulk splenoctyes from IκBαM.MR-infected Bcl2 mice had a similar 50-fold reduction in frequency of cells reactivating virus compared to the frequency of cells harboring viral genome (Figure 6B and summarized in Table 2). The reactivation of purified CD19^+^ B cells were reduced approximately 3- to 4-fold compared to bulk splenocytes, a decrease that may reflect the time and manipulation required to purify these cells. The frequency of unsorted and CD19^+^ splenocytes recovered from IκBαM.1-infected mice was approximately 50-fold lower than observed with the marker rescue virus (Figure 6B and Table 2). Taken together, these data confirm the major defect in latency establishment by IκBαM.1 and argue that inhibiting NF-κB regulation of Bcl-2 expression alone does not account for the phenotype of the IκBαM-expressing viruses. In addition, since no difference in the efficiency of spontaneous virus reactivation was observed with latently infected splenocytes harvested from Bcl-2 transgenic mice compared to C57Bl/6 mice, this indicates that survival of latently infected B cells in culture does not play a significant role in dictating the observed frequency of splenic B cells reactivating virus.

**Table 2:**
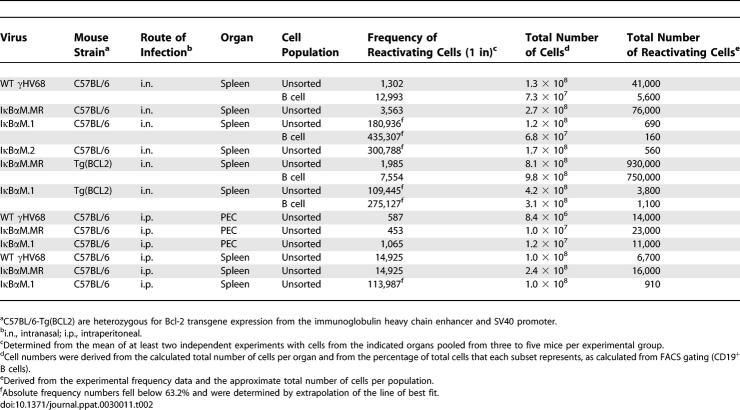
Frequencies of Cell Populations Reactivating Virus at 15 to 16 dpi

Extending the analysis of latency in Bcl-2 transgenic mice, we observed at later times postinfection that there was a slightly greater defect in latency in Bcl-2 trangenic mice infected with IκBαM.1 (one in 1,983) compared to IκBαM.MR (one in 295) (Figure 6C) than observed in C57BL/6 mice. This defect was also maintained at 235 dpi with a nearly 10-fold difference (*p* = 0.0181) between the frequency of viral genome–positive splenocytes from mice infected with IκBαM.1 (one in 6,139) and IκBαM.MR (one in 695) (Figure 6D). In addition, these data are consistent with the interpretation that there is a steady rate of decay in the B-cell reservoir over time for both IκBαM.1 and IκBαM.MR once the initial contraction phase between 16 and 41 dpi occurs. Taken together, the lack of complementation by Bcl-2 at any time point after infection indicates that NF-κB signaling likely provides a role distinct from peripheral B-cell survival during γHV68 latency.

### Inhibition of NF-κB Activation Reduces Latency in Lung B Cells

Recent reports indicate that lung B cells are infected early after virus inoculation and may represent a long-term reservoir for viral persistence [29,48,49]. As B cells are critical for acute replication and seeding of the spleen at early times after infection [21,22], we hypothesized that the approximately 50-fold reduction of splenic latency in mice infected with IκBαM.1 might be due to a reduction in seeding of the spleen from γHV68-infected lung B cells. Indeed, acute virus replication in the spleen at 9 and 12 dpi demonstrated that IκBαM.1 grew more poorly in the spleens of infected mice compared to IκBαM.MR (Figure 2E), even though IκBαM.1 exhibited WT replication kinetics in the lungs (Figure 3D). This suggested the possibility of a defect in trafficking of infected B cells from the lung to the spleen and led us to investigate the ability of IκBαM.1 to establish latency in unsorted lung cells and B cells (CD19^+^) at 16 dpi.

**Figure 7:**
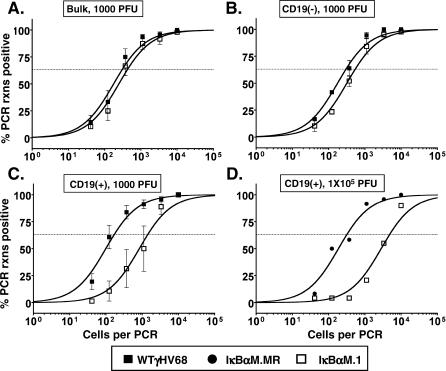
IκBαM.1-Infected Mice Have Decreased Latency in Lung B Cells 16 dpi after Intranasal Infection Bulk lung cells, or lung cell subsets, were harvested from infected C57BL/6 mice at 16 dpi and analyzed by limiting-dilution viral genome PCR as described in Materials and Methods. (A) Frequency of unsorted, bulk lung cells harboring viral genomes. (B) Frequency of CD19^−^ non–B cells harboring viral genomes. Using a PE-conjugated antibody to the pan–B-cell marker CD19 (CD19-PE), splenic B cells were separated into B-cell (CD19^+^) and non–B-cell (CD19^−^) populations by magnetic activated cell sorting. Postsort FACS analysis indicated that the mean purities for CD19^−^ cells were 97.2 ± 2.4% for WT γHV68 and 98.7 ± 0.9% for IκBαM.1. (C) Frequency of subsets enriched for CD19^+^ B cells harboring viral genome. Subsequent to magnetic separation, postsort FACS analysis indicated that the mean purities for CD19^+^ cells were 82.3 ± 0.5% for WT γHV68 and 82.0 ± 1.3% for IκBαM.1. Given that in presort FACs analysis CD19^+^ cells comprised approximately 9% of the lung cell suspension, magnetic separation generated a 9-fold enrichment of CD19^+^ B cells. The data shown represent three independent experiments with spleen cells pooled from 12 to 15 mice per experimental group. The frequency of splenocytes and CD19^+^ B cells that harbored viral genome in mice infected with IκBαM was significantly different (*p* = 0.0316) from that of mice infected with WT viruses. (D) Frequency of FACS sorted CD19^+^ B cells harboring viral genomes in the lungs of mice infected with 1 × 10^5^ PFU of IκBαM.MR or IκBαM.1. Postsort FACS analysis indicated that the purity of the CD19^+^ cell population was 86% for IκBαM.MR and 93% for IκBαM.1. The data shown represent a single experiment with 15 mice per group. Curve fit lines were derived from nonlinear regression analysis, and symbols represent the mean percentage of wells positive for virus (viral DNA) ±SEM.

At 16 dpi, there were nearly equivalent frequencies of viral genome–positive unsorted lung cells in mice infected with IκBαM.1 (one in 422) and WT γHV68 (one in 298) (Figure 7A). CD19^+^ (B cells) and CD19^−^ (non–B cells) were enriched from the bulk lung cells and analyzed for the presence of viral genome by limiting-dilution PCR. Non–B cells harbored nearly identical frequencies of viral genomes (Figure 7B). However, as shown in Figure 7C, lung B cells from IκBαM.1 (one in 1,447)-infected mice had significantly decreased levels of viral genomes compared to mice infected with WT virus (one in 138) (*p* = 0.0316). This represents an approximately 90% reduction in the frequency of viral genome–positive lung B cells in mice infected with the IκBαM.1 recombinant virus (Table 1). Because it has been shown that increasing the dose of virus inoculation can overcome the defect in latency establishment of a virus containing a stop mutation in the latency-associated M2 gene [44], we increased the dose of virus 100-fold to 1 × 10^5^ PFU and examined infection of lung B cells by limiting-dilution PCR analysis of FACS-sorted CD19^+^ cells (B cells). Notably, lung B cells from mice infected with IκBαM.1 exhibited a 15-fold reduction in the establishment of latency, even with the higher inoculating dose (Figure 7D). These data indicate that mice infected with a virus that inhibits NF-κB activation exhibit a significant reduction in the establishment of latency in B cells of the lung, a phenotype which could not be overcome by increasing the inoculating dose of virus. It should be noted that the magnitude of the defect observed with the IκBαM-expressing virus in the lungs (10- to 15-fold) was less than that observed in the spleen (approximately 50-fold), suggesting that in addition to playing a role in the establishment of B-cell latency, NF-κB activation also plays a role in subsequent events such as trafficking of latently infected B cells from the lung to the spleen and subsequent establishment of splenic latency.

### Decreased B-Cell Activation in the Lungs and Spleen of IκBαM-Infected Mice

NF-κB is well known for its role in recruiting immune cells by driving the expression of inflammatory cytokines, chemokines, adhesion molecules, and inflammatory mediators. To determine whether inhibition of NF-κB signaling in IκBαM-infected mice impacts the global immune response, we assessed the phenotypes of B and T cells in the lungs and spleens 16 and 42 dpi with 1,000 PFU of IκBαM.1 and WT virus. The number of B cells (8.2% versus 12.4%, *p* = 0.0548), CD4^+^ T cells (9.6% versus 12.9%, *p* = 0.1065), and CD8^+^ T cells (12.9% versus 18.1%, *p* = 0.0268) present in the lungs of IκBαM.1-infected mice was slightly diminished at 16 dpi compared to WT virus–infected mice (Table 3; unpublished data). However, there was no significant difference in the activation state of T cells as determined by upregulation of CD11a (unpublished data). Notably, the relatively small difference in the number of B cells present in the lungs is unlikely to contribute significantly to the observed decrease in establishment of B-cell latency in mice infected with the IκBαM.1 virus.

**Table 3:**
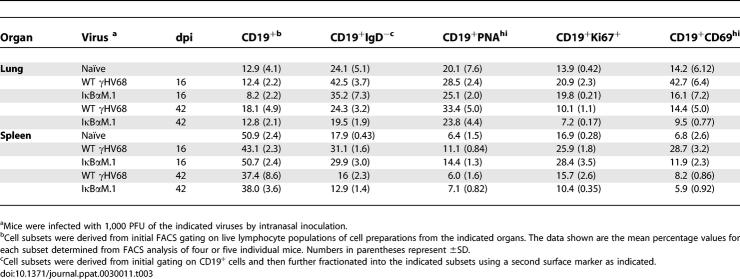
Analysis of B-Cell Phenotype by Surface Antigen Markers in Infected C57BL/6 Mice

**Figure 8:**
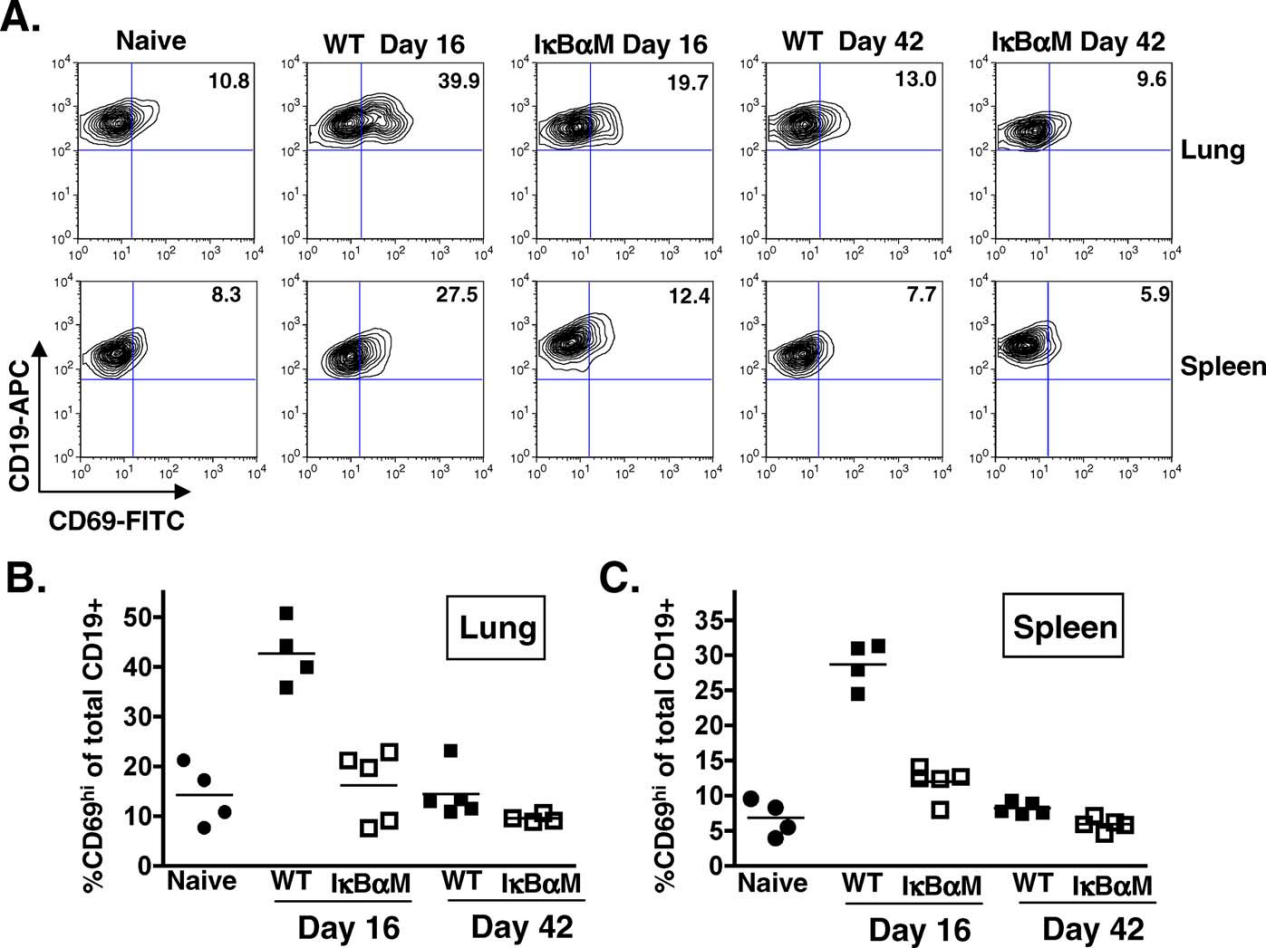
Activated B Cells Are Decreased in the Lungs and Spleens of IκBαM-Infected Mice Cells were prepared from the lungs and spleens harvested from four uninfected naïve mice or from four or five mice that were infected 16 and 42 d prior to harvest with 1,000 PFU of WT γHV68 or IκBαM.1. Cells from individual mice were surface stained with anti-CD19 conjugated to allophycocyanin and anti-CD69 conjugated to FITC and analyzed by flow cytometry. (A) Flow cytometric dotplots of stained cells from the lungs (upper panel) and spleens (lower panel). Values shown in the upper right quadrant of each dotplot are the percentages of CD19^+^ cells that express the CD69 activation marker in a representative experimental sample. (B) Each point in the scatterplot represents the percentage of CD19^+^CD69^hi^ cells from the lung of a single mouse; the bar represents the mean percentage. The percentage of CD19^+^CD69^hi^ cells in the lungs of mice infected with IκBαM.1 was significantly different from that of mice infected with WT γHV68; *p* = 0.0007 at 16 dpi. (C) Each point in the scatterplot represents the percentage of CD19^+^CD69^hi^ cells from the spleen of a single mouse; the bar represents the mean percentage. The percentage of CD19^+^CD69^hi^ cells in the spleens of mice infected with IκBαM.1 was significantly different from that of mice infected with WT γHV68; *p* < 0.0001 at 16 dpi and *p* = 0.0042 at 42 dpi.

To assess whether there were phenotypic differences between the B-cell populations in IκBαM.1- and WT virus–infected mice, CD19^+^ lung and splenic B cells were analyzed for markers of isotype-class switching (IgD^−^), germinal center participation (PNA^hi^), proliferation (Ki67^+^), and activation (CD69^hi^) at 16 and 42 dpi (Table 3). The total number of B cells and the frequency of IgD and PNA^hi^ CD19^+^ B cells were slightly lower in the lungs of IκBαM.1-infected mice compared to control virus. The proliferation of B cells in the lungs of infected mice was also examined by the measuring bromodeoxyuridine incorporation. Mice were administered bromodeoxyuridine in their drinking water from 8 to 16 dpi; no significant difference in the frequency of B cells incorporating bromodeoxyuridine was observed in IκBαM.1 virus–infected mice compared to IκBαM.MR virus–infected mice (unpublished data). The only significant difference in B cells from mice infected with IκBαM.1 compared to WT virus at 16 dpi was a decreased frequency of CD19^+^CD69^hi^ B cells (Figure 8A). In both the lung (*p* = 0.0003) and spleens (*p* < 0.0001), there was an approximately 3-fold reduction in CD69^hi^ B cells in IκBαM.1-infected mice compared to mice infected with IκBαM.MR at day 16 (Figure 8B). By 42 dpi, the frequency of CD69^hi^ B cells in mice infected with either WT virus or the IκBαM.1 virus was not significantly different from that observed in uninfected mice (Figure 8). Since CD69 is known to play an important role in lymphocyte trafficking, the difference in CD69 levels between IκBαM.1 and WT virus may contribute to the more severe latency phenotype observed in splenic B cells compared to lung B cells at 16 dpi [50]. However, it is important to note that (i) only approximately 1% of B cells at day 16 are γHV68 latently infected and thus the vast majority of the CD69^+^ B cells are not virus infected and (ii) we currently do not have the necessary tools to assess the status of CD69 expression on latently infected B cells.

### Intraperitoneal Inoculation of IκBαM.1 Partially Rescues the Defect in EStablishment of Splenic Latency

**Figure 9:**
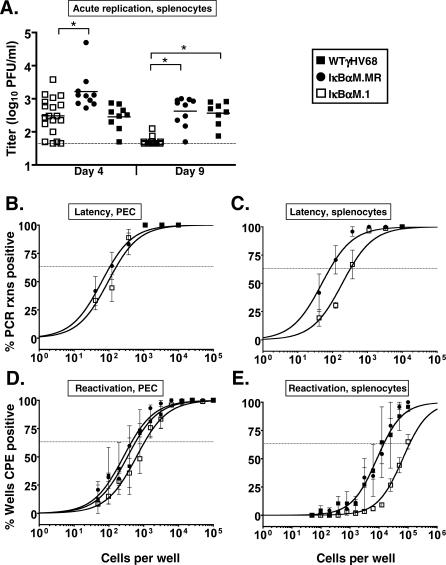
Route- and Tissue-Dependent Requirement for NF-κB C57Bl6 mice were infected with 1,000 PFU by the intraperitoneal route of inoculation with the indicated viruses. (A) On 4 and 9 dpi, spleens were harvested, disrupted, and titered on NIH 3T12 cells. The data are compiled from two (IκBαM.MR and WT) or four (IκBαM.1) experiments with four or five mice per group. Asterisks denote that the acute splenic titers for IκBαM.1 differed significantly from IκBαM.MR on day 4 (*p* = 0.0036) and from IκBαM.MR (*p* < 0.0001) and WT (*p* < 0.0001) on day 9, as determined by the Mann-Whitney nonparametric test. (B) Frequency of PECs harboring viral genomes. (C) Frequency of unsorted, bulk splenocytes harboring viral genome. (D) Frequency of PECs reactivating virus. (E) Frequency of splenocytes reactivating virus. For both limiting-dilution assays, curve fit lines were derived from nonlinear regression analysis, and symbols represent the mean percentage of wells positive for virus (viral DNA or cytopathic effect) ±SEM. The data shown represent three to five independent experiments with cells pooled from five mice per experimental group.

Although increasing the dose of virus used to inoculate mice via the intranasal route did not rescue the defect in establishment of B-cell latency in the lungs observed with the IκBαM.1, we addressed whether altering the route of inoculation might have an impact on the establishment of latency. The kinetics of IκBαM.1 replication in the spleen was compared to IκBαM.MR and WT virus following intraperitoneal inoculation of mice with 1,000 PFU of virus. At days 4 and 9 after intraperitoneal inoculation with 1,000 PFU, spleens were harvested and titered. Mice infected with IκBαM.1 had comparable replication kinetics to WT virus at 4 dpi and slightly lower acute viral titers in the spleen compared to those infected with IκBαM.MR (Figure 9A). Surprisingly, there was a significant reduction in detectable virus replication in the spleens of mice infected with IκBαM.1 at 9 dpi compared to IκBαM.MR (*p* < 0.0001) and WT virus (*p* < 0.0001) (Figure 9A). This indicates that IκBαM.1 is impaired in the duration of acute replication compared to IκBαM.MR and WT virus in the spleens of mice infected by the intraperitoneal route. Notably, a shortened period of acute phase replication has previously been observed for some other γHV68 mutants that are not impaired in the establishment of splenic latency [51–53], indicating that a decreased duration of acute replication in the spleen does not directly correlate with the levels of splenic latency that are established following intraperitoneal inoculation. Since no defect in virus replication in the lungs was observed following the same dose of virus administered via intranasal inoculation (see Figure 3B), this argues that there are distinct requirements for acute virus replication at different anatomical sites.

To assess the establishment of viral latency, we initially examined latency in peritoneal exudate cells (PECs). PECs were harvested from infected mice at 16 dpi and the frequency of cells harboring virus was determined by limiting-dilution PCR. The frequency of viral genome–positive PECs in mice infected with IκBαM.1 (one in 166 cells) was nearly equivalent to the frequency of observed in mice infected with IκBαM.MR (one in 108 cells) (Figure 9B). The frequency of PECs that reactivated virus from mice infected with IκBαM.1 (one in 1,065), IκBαM.MR (one in 453), and WT (one in 587) viruses was nearly identical and was approximately 5-fold lower than the frequency of viral genome–positive cells (Figure 9D). As macrophages comprise the majority of the γHV68-infected cells in the peritoneum of mice [26], these results indicate that NF-κB activation is not likely required for the establishment of latency, or for reactivation, from latently infected macrophages. Importantly, we did not detect any preformed infectious virus in the disrupted PEC samples (unpublished data).

Finally, we assessed the consequences of inhibiting NF-κB activation on the establishment of splenic latency at day 16 post intraperitoneal inoculation (Figure 9C). Splenocytes from mice infected with IκBαM.1 were found to harbor viral genome at a frequency of one in 327 splenocytes, a 4-fold decrease in establishment compared to a frequency of one in 85 splenocytes upon IκBαM.MR infection (*p* = 0.0437). The frequency of splenocytes reactivating virus from IκBαM.MR- and WT virus–infected mice was nearly identical (one in 14,925), while the frequency of splenocytes reactivating virus from IκBαM.1-infected mice was estimated to be one in 113,987, an approximately 8-fold decrease in the levels of reactivating virus compared to IκBαM.MR-infected mice (Figure 9E). The latter results are in close agreement with the defect in the establishment of viral latency. Importantly, the defect in establishment of splenic latency following intraperitoneal inoculation of the IκBαM recombinant virus was significantly less than that observed following intranasal inoculation (see Figure 4), which may be related to differences in virus trafficking, target B-cell populations, and/or transgene expression (see Discussion).

## Discussion

Here we report several novel findings regarding the role of NF-κB signaling in gammaherpesvirus pathogenesis. First, γHV68 activated NF-κB in the course of a productive infection, yet did not require NF-κB signaling for productive replication in murine fibroblasts in tissue culture or during acute replication in the lung in vivo. Second, NF-κB activation was required for the efficient establishment of latency in B cells of the lung and spleen, and this decrease in latency correlated with a reduction of CD69^hi^CD19^+^ B cells following intranasal inoculation. Third, we found no evidence for the contribution of virus reactivation and reseeding of latency to the slower rate of contraction of the latently infected reservoir in the spleen (observed between 2 and 6 wk postinfection) of mice infected with the IκBαM-expressing virus versus WT γHV68. Fourth, constitutive expression of the antiapoptotic Bcl-2 protein in B cells did not rescue the defect in establishment of splenic latency observed with the γHV68-IκBαM–expressing virus at early or late times postinfection. Finally, upon intraperitoneal inoculation, NF-κB activation is required for sustained acute phase replication, yet its inhibition resulted in a less severe impairment in the establishment of splenic latency. Taken together, these data demonstrate that NF-κB activation plays an important role in the establishment of latency by γHV68.

### NF-κB Is Activated but Not Required for γHV68 Replication

Overexpression of the NF-κB subunit p65 inhibits the progression of γHV68 lytic replication in 293T cells [33]. The inhibition by p65 may relate to its ability to inhibit transactivation of promoters by γHV68 replication and transcription activator (RTA), as reported for KSHV RTA [33,54]. Thus, a virus that inhibits NF-κB and does not antagonize RTA might be predicted to have faster replication kinetics, similar to a γHV68 mutant that expresses RTA constitutively [55]. Indeed, the inability to detect γHV68–IκBαM replication in the spleen 9 d after intraperitoneal inoculation could be attributed to a more rapid peak of acute phase replication (Figure 9A). However, unlike the results reported with a dysregulated RTA-γHV68 mutant [55], IκBαM had no significant alteration in the kinetics of growth in fibroblast cells in culture and did not exhibit heightened peak titers in the lungs after intranasal inoculation (Figure 3). These data highlight a tissue-dependent requirement for NF-κB during productive replication but do not support an increase in lytic kinetics as an explanation for the substantial decrease in splenic latency upon intranasal infection with γHV68-IκBαM.

### Role of NF-κB in the Efficient Establishment of Splenic Latency

CD40L, BAFF, and lymphotoxin are costimulatory signals provided by dendritic cells and T cells in germinal centers that are required to drive NF-κB activation for B-cell survival upon activation and differentiation [56]. Given that γHV68 establishes latency in B cells bearing surface markers characteristic of cells proliferating and participating in germinal center reactions [2,30,31], NF-κB is likely required upon activation for proliferation of the infected cells. Examination of splenocytes determined that infection with γHV68-IκBαM resulted in a substantial (97%) inhibition in the establishment of latency 16 dpi in comparison to WT virus (Figure 4 and Table 1). We also determined the frequency of latently infected cells in B-cell and non–B-cell subpopulations; γHV68-IκBαM had a larger deficit in splenic establishment in B cells (98% reduction) compared to non–B cells (83% reduction).

The lack of absolute ablation of latency upon infection with IκBαM might reflect (i) the utilization of alternative NIK/IKK1-mediated NF-κB activation, (ii) the establishment of virus infection in cell populations that do not rely on NF-κB, and/or (iii) inadequate expression of the IκBαM transgene; the HCMV immediate-early promoter might not drive sufficient levels of IκBαM expression in some cells or cell populations (as such, the residual latency observed at 16 dpi might simply reflect a population of cells in which NF-κB activation was not suppressed). Regardless, utilization of the transdominant IκBαM clearly had a substantial impact on the ability of γHV68 to establish latency in vivo.

In a mixed CD40^+^CD40^−^ bone marrow chimeric system, both CD40-deficient and CD40-sufficient B cells were found to harbor viral genomes at 14 dpi at similar frequencies [3]. However, over time, latently infected CD40-deficient B cells were lost. This is consistent with an interpretation for a requirement for CD40-mediated signaling and participation in germinal centers of infected mice for long-term maintenance of γHV68 B-cell latency [3]. In contrast to the failure to maintain latency in CD40-deficient B cells at later timepoints [3], γHV68-IκBaM–infected B cells exhibit a defect in the establishment of latency at 16 dpi. This indicates that activation of NF-κB plays a critical role in addition to, and prior to, the engagement of infected cells with CD40L in germinal center reactions. Thus, the impairment in the establishment of splenic latency at 16 dpi in mice infected with the recombinant γHV68-expressing IκBαM strongly suggests that NF-κB activation mediated by the classic pathway is essential for the efficient establishment of latency at early time points.

Upon encountering antigen, NF-κB functions in mature B cells as a survival factor by mediating the upregulation of several antiapoptotic proteins, including A1, Bcl-2, and Bcl-xL [7,57]. One explanation for the large defect in latency establishment is that the inhibition of NF-κB results in the loss of expression of antiapoptotic proteins, such as Bcl-2 or Bcl-xL, leading to cell death. Bcl-2 knockout mice lack mature B cells in the periphery, demonstrating a role for Bcl-2 in peripheral B-cell survival [58]. Transgenic mice with targeted expression of Bcl-2 in B cells are characterized by larger numbers of B cells and reduced apoptosis in germinal centers, leading to an accumulation of memory B cells with a low frequency of somatic hypermutation [59–61]. Bcl-2 and Bcl-xL reduce apoptosis in resting and activated crel^−/−^ and nfkb1^−/−^ mature B cells but do not restore proliferative responses in response to mitogenic stimuli [62,63]. Although Bcl-2 complemented BAFF^−/−^ mice for peripheral B-cell survival, it did not restore differentiation and marginal zone B cells [64]. Similarly, BAFF-R–deficient mice expressing the Bcl-2 transgene remain defective in the formation of lymphoid follicles and germinal centers [65]. Taken together, Bcl-2 transgenic mice might be expected to rescue a latency defect for γHV68-IκBαM if NF-κB is required for survival in the periphery but would be unlikely to rescue a defect in activation-induced proliferation and participation in germinal center reactions.

As shown here (see Figure 6), in the presence of both the endogenous virally encoded bcl2 homolog (M11) and constitutive expression of the cellular Bcl-2 in B cells, the deficit in latency establishment was still observed. γHV68-IκBαM exhibited a 99% reduction in latency establishment in these mice (both in bulk splenocytes and in purified B cells) compared to IκBαM.MR at 16 dpi (Table 1). γHV68-IκBαM viral load remained 10-fold lower than IκBαM.MR at 6 wk and even through 7 mo after infection. The lack of latency restoration for γHV68-IκBαM in the Bcl-2 transgenic mice indicates that NF-κB plays a critical role(s) in addition to cell survival.

The complementation of cell survival, but not proliferation, in response to mitogenic stimulation by the bcl-2 transgene in c-rel^−/−^ B cells indicates that NF-κB likely functions to promote cell cycle progression in a Bcl-2–independent manner [63]. c-rel^−/−^ B cells fail to enter S phase upon B-cell receptor activation, a block in cell cycle progression that is attributed to the failure to induce the transcription of G1 cyclins D3 and E, which in turn leads to a loss of cdk activity and delayed pRB phosphorylation [66]. NF-κB activation likely mediates important roles in cell cycle progression that are required during the proliferative expansion of γHV68-infected B cells in the spleen [31,32].

### Maintenance of γHV68 Latency

An examination of the maintenance of long-term latency at 6 wk postinfection demonstrated, as previously observed, a significant contraction in splenic latency from 16 to 42 dpi for WT and marker rescue, in both C57BL/6 and Bcl-2 transgenic mice. However, a similar magnitude of contraction of splenic latency was not observed in γHV68-IκBαM–infected mice. The end result is that the latency defect of the γHV68-IκBαM virus is diminished at later times postinfection. Similar observations have been made for other γHV68 mutants, including viruses with targeted mutations in the M2, M4, gp150, and viral TK genes [44,45,51,67,68], all of which exhibit severe latency establishment phenotypes at day 16 postinfection but less substantial latency phenotypes at later times postinfection. The slower decline in splenic latency for γHV68-IκBαM might be attributed to (i) a delay in virus (or latently infected cell) trafficking from the lung to the spleen, (ii) a contribution of virus (or latently infected cells) from nonsplenic reservoirs of latency that supplements infected cell turnover in the spleen, (iii) direct infection of a more stable, nonproliferating reservoir of cells (e.g., memory B cells), (iv) an accumulation of virus in one particular subset of cells that compensates for loss in another, and/or (v) altered trafficking of latently infected cells from the spleen to other sites (egress). Cidofovir treatment of B-cell–deficient mice between 16 and 42 dpi has been demonstrated to reduce viral latency in PECs and splenocytes after intraperitoneal infection [46,47]. In contrast, utilizing the same treatment regimen in C57BL/6 mice infected with the IκBαM or IκBαM.MR virus, there was no change in the levels of viral latency in the spleens of mice following antiviral therapy from 16 to 39 dpi. These data are consistent with the interpretation that virus reactivation and seeding of the spleen from alternative reservoirs does not contribute to splenic latency at 6 wk postinfection. It is possible that the IκBαM-expressing γHV68 may directly infect a nonproliferative stable cell reservoir, such as memory B cells, that is detected at all time points.

### Route-Specific Requirements for NF-κB Activation in the Establishment of Splenic Latency

We have previously observed route-dependent phenotypes for a γHV68 mutant in which the M2 gene has been disrupted [44,51]. Similarly, when mice were infected via intraperitoneal inoculation, a distinctly different phenotype of the γHV68-IκBαM was observed. By this route, acute phase replication at 9 dpi was nearly undetectable compared to WT or MR, yet these altered kinetics did not substantially affect the establishment of latency in the PEC or splenocytes by the IκBαM virus at 16 dpi. Instead, the defect in the establishment of splenic latency was more modest than observed following intranasal inoculation (compare Figures 4 and 9).

A disconnect between lytic replication and latency establishment following intraperitoneal inoculation has been reported for other γHV68 mutants (e.g., a γHV68 mutant lacking 9.5 kb at the left end of the genome [53], a γHV68 mutant containing a stop mutation in the M2 ORF [51], or a γHV68 mutant containing an insertion of a LacZ expression cassette in the M1 ORF [52]). Interestingly, mice lacking B cells do not maintain acute replication of WT γHV68 in the spleen at day 9 following intraperitoneal infection, yet the spleens do harbor significant levels of latently infected cells at later timepoints [22,43]. This indicates a role for B cells in prolonging the acute phase of lytic replication, perhaps via reactivation and seeding permissive cells in the spleen. However, it is notable that B cells appear dispensable for the establishment of latency in other cell types in the spleen (e.g., dendritic cells and macrophages) [22]. Further characterization of the kinetics of mutant virus replication and the induction of the host immune response are required to understand the basis for the more rapid clearance of acute virus replication in the spleen for the IκBαM-expressing virus. Regardless of the mechanism for the shortened acute phase of replication, we can infer that the virus has accessed the latent reservoir of the spleen before 9 dpi after intraperitoneal inoculation.

Following intraperitoneal inoculation, γHV68 did not require NF-κB activation for establishment of latency, or reactivation from latency, in PECs. Since macrophages are the major component of PECs [26], this suggests that NF-κB activation is dispensable for establishment of γHV68 latency in macrophages. This is consistent with the observation that the frequency of viral genome–positive non–B cells in the lungs at day 16 was also largely unaffected by inhibition of NF-κB activation (see Figure 7). In contrast to PECs, there was a slight, but significant, decrease in latency establishment in the spleens of mice following intraperitoneal infection with IκBαM-γHV68. This phenotype also was apparent in a frequency analysis of latency in sorted B-cell populations (unpublished data). The mild phenotype upon intraperitoneal inoculation with IκBαM-γHV68 compared to the approximately 50-fold decrease in splenic latency upon intranasal infection indicates that there are critical mechanistic differences with respect to seeding splenic latency between these routes of inoculation. The analysis of splenic latency in IκBαM-γHV68–infected mice following intraperitoneal inoculation suggests the establishment of a form of B-cell latency that does not require an active role for the virus in driving activation and proliferation of these infected splenic B cells, similar to phenotypes uncovered for M2- and v-bcl2–null viruses [44,46,51,69]. Further investigation of splenic latency following intraperitoneal inoculation is required to fully understand the observed differences in establishment of splenic B-cell latency.

### Requirement for NF-κB Activation in the Establishment of Viral Latency in Lung B Cells

Following intranasal inoculation, there is a significant impact of inhibiting NF-κB activation on establishment of latency in the spleen, but this phenotype is reduced substantially upon the more direct and permissive intraperitoneal route of inoculation (compare Figures 4 and 7). This raises the possibility that altered trafficking of latently infected cells to the spleen also contributes to the splenic latency phenotype observed at day 16 with the IκBαM-expressing virus following intranasal inoculation. Interestingly, although splenic latency is established in B-cell–deficient mice after intraperitoneal inoculation, there is a defect in the establishment of latency in the spleen following intranasal inoculation of these mice [21,24]. Adoptive transfer of B cells into B-cell–deficient mice infected with γHV68 restored latency in the spleen [23]. In addition, it has previously been reported that intranasal infection of mice with replication defective mutants (ORF31- or ORF50-null viruses), resulted in virus infection of B cells in the lung, yet these viruses were not detected in the spleen [48,49]. Taken together, the seeding of the spleen may result from reactivation of latently infected B cells that traffick from the lungs to the spleen, thereby seeding acute virus replication in the spleen and the subsequent establishment of splenic latency. This led us to investigate NF-κB–mediated functions in the lung, the site of primary infection.

As shown here, the IκBαM-expressing γHV68 was substantially impaired in the establishment of latency in lung B cells (see Figure 7), where a 90% reduction in latency in this particular cell subset was observed. It is also notable that increasing the inoculating dose of virus 100-fold did not ameliorate this defect in B-cell latency in the lungs. The defect in establishment of B-cell latency in the lungs (Figure 7), coupled with both a slight diminishment of acute replication in the spleen (Figure 3E) and a substantial defect in the establishment of latency in the spleen (Figure 4), suggests that the efficient seeding of γHV68 latency in the spleen following intranasal inoculation of virus requires the egress of latently infected B cells from the lung.

### Reduction in Activated B Cells upon NF-κB Inhibition

The absence of heightened lytic replication or viral load in latency reservoirs in mice infected with IκBαM-expressing γHV68 demonstrates that the host is able to mount an effective immune response (i.e., this method of targeted NF-κB inhibition only in virus-infected cells does not appear to result in any gross immune dysfunction). However, the requirement of NF-κB activation for the efficient establishment of lung B-cell latency could reflect, in part, a role for this host cell factor in mediating inflammatory cytokine production by infected cells that could lead to alterations in the recruitment, activation, and/or subsequent trafficking of infected cells to the spleen. In an examination of the profile of lymphocytes in both the lungs and spleens at early and late times after infection, we failed to observed any significant differences in the numbers of B or T cells recruited to the lungs or present in the spleens of mice infected with the IκBαM-expressing γHV68. The number of PNA^hi^ and IgD^−^ cells were slightly lower for IκBαM-infected mice. The frequency of proliferating cells at 16 dpi, as detected by the Ki67 nuclear proliferation antigen (Table 3), and confirmed by bromodeoxyuridine incorporation from 8 through 16 dpi (unpublished data), was not dramatically reduced. This contrasts with the nearly 3-fold decrease in the activation of B cells in the lungs and spleens at 16 dpi as determined by the presence of the CD69 activation marker, which correlated with the reduction in viral load in the B cells of the lung and spleen.

It is known that γHV68 infection results in polyclonal activation of B cells, characterized by an upregulation of the CD69 activation marker and an increase in nonspecific antibody production, a process that is dependent on CD4^+^ T cells [43,70,71]. A reduction in CD69 activation has been correlated to both a decrease in splenomegaly and splenic latency in mice infected with γHV68 mutants deficient in vbcl-2 [69], containing a large insertion in the M3 gene [72], or a modification linking the ORF73 product to a CD8–T-cell epitope [73]. The lack of an acute replication defect for the IκBαM-expressing γHV68, as with these other mutants after intranasal infection, substantiates the conclusion that CD69 activation correlates with differences in the levels of splenic latency and extends that correlation to latency in B cells at the primary site of infection. It is very unlikely that each of these mutated ORFs has a direct role in CD69 upregulation, as acute titers were normal for each mutant. Considering the disconnect between the large numbers of B cells activated and the small fraction that is virus infected, there may be a factor secreted by latently infected B cells that activates neighboring B cells in a paracrine manner. Nonspecific B-cell activation might represent a mechanism by which the virus drives B-cell participation in germinal center reactions to increase the likelihood of gaining access to the long-lived memory B-cell reservoir.

NF-κB activation via the classic signaling pathway drives the expression of a multitude of proinflammatory molecules, including the cytokines TNF, interleukin (IL)-1, and IL-6; chemokines RANTES (regulated on activation, normal T-cell expressed and secreted), macrophage inflammatory protein-1a, and membrane cofactor protein-1, and the inflammatory mediators inducible nitric oxide synthase and cyclooxygenase 2 (COX-2) (reviewed in [74]). These inflammatory molecules are detected in lung lavage during acute γHV68 replication [75–77] and might be altered upon infection with the IκBαM-expressing virus. Inducible nitric oxide synthase^−/−^ mice have been reported to exhibit no alteration in immune clearance or splenomegaly upon γHV68 infection [78]. NF-κB also drives the expression of cellular COX-2, an enzyme that catalyzes the production of the inflammatory prostaglandin E_2_. COX-2 is induced upon γHV68 infection in vitro, and the inhibition of COX-2 with the nonsteroidal anti-inflammatory drug NS-398 reduced viral protein production and virion production in vitro [79]. However, this group reported that the targeting of COX-2 did not translate into an inhibition of acute replication in the lungs of mice upon treatment with NS-398 [79]. IL-6 is detected in the lungs and lymph nodes of infected mice and is produced upon in vitro stimulation of lymphocytes from infected mice [80,81]. However, there was no difference in virus growth, in establishment of latency, or in the immune response to the virus in IL-6^−/−^ mice [81,82]. The inflammatory response to γHV68 is multifaceted, involving the contribution of proinflammatory molecules that are produced by the heterogeneous population of infected cells and the multiple immune cell types that infiltrate and are activated at the site of infection [75]. Thus, the lack of a substantial phenotype upon the knockout of single NF-κB–regulated inflammatory components is not altogether unexpected. In future studies, the characterization of the cytokine and chemokine responses to infection by IκBαM-expressing γHV68 might reveal alterations of biological relevance.

### Manipulation of NF-κB Activation by Gammaherpesviruses

A number of gammaherpesvirus gene products have been shown to modulate NF-κB activity. EBV LMP-1 and the NF-κB modulatory proteins of KSHV, vIL-6, K15, K1, and vFLIP are not encoded by γHV68 and, as such, are not candidates for mediating NF-κB activation during establishment of γHV68 latency. The γHV68 viral G protein–coupled receptor (vGPCR; encoded by ORF 74 and a homolog of the KSHV vGPCR) is a weak inducer of NF-κB activation in a ligand-dependent manner [83,84]. The expression of the chemokines RANTES and macrophage inflammatory protein, which mediate vGPCR activation in cell culture, are detected in the lungs of γHV68-infected mice [85], raising the possibility that vGPCR mediated induction of NF-κB during the acute phase of virus replication in the lungs. However, the significance of such modulation on the establishment of latency is unclear, since a stop mutation in ORF74 manifests as a defect in reactivation, not latency establishment in vivo [83,86].

Given the importance, as shown here, of NF-κB activation for establishment of latency in vivo, it is possible that initial activation of NF-κB (e.g., through virus binding and entry) may serve to promote establishment of a latent infection in the appropriate cellular context prior to expression of latency-associated viral genes that activate NF-κB. For example, in the case of EBV infection of primary B cells, NF-κB is activated upon the binding of the viral glycoprotein gp350 to the cell surface [87]. It is not known whether binding of the γHV68 homolog of EBV gp350, gp150, is involved in the initial activation of NF-κB during γHV68 infection of permissive cells. Regardless, the phenotype of the gp150-null virus is distinct from the IκBαM phenotype [88], making it unlikely that gp150 plays a critical role in activating NF-κB during the establishment of viral latency. It seems very likely that γHV68 encodes other, as-yet-unidentified, gene products that modulate the activation of NF-κB to manipulate the cell and the host microenvironment and thereby optimize conditions for viral persistence.

### Conclusions

We have shown here that NF-κB signaling is critical for efficient establishment of viral latency following intranasal inoculation of γHV68 pathogenesis and, as such, likely represents a key host factor that is manipulated by the virus to establish latency in B cells. To our knowledge, this is the first report to identify a host transcription factor required for the efficient establishment of gammaherpesvirus latency in vivo. As NF-κB signaling is intimately involved in inflammation and B-cell biology, future studies aim to determine the role of specific NF-κB subunits and signaling pathways involved in the establishment of latency, cytokine production, trafficking, proliferation, and participation of B cells in germinal center reactions. Further characterization of the molecular mechanisms by which γHV68 modulates NF-κB activation is critical to understanding virus–host interactions that transpire, ultimately allowing the virus to gain access to the long-lived memory B-cell reservoir. We anticipate that γHV68 latency in B cells closely parallels EBV infection in humans, which involves virus-driven B-cell proliferation and differentiation. In the case of EBV infection, it is thought that the ability of the virus to manipulate B-cell differentiation places the host at greater risk for lymphoproliferative disorders and lymphoma. Furthermore, modulation of NF-κB activation by EBV is clearly critical to these processes. Exploiting γHV68 infection of mice as a model system may lead to critical insights into the role of gammaherpesviruses in lymphomagenesis.

## Materials and Methods

### Viruses and tissue culture.

γHV68 WUMS (American Type Culture Collection VR1465, http://www.atcc.org) was the WT virus. Virus passage and titer were performed as previously described [22]. NIH 3T12 cells and MEFs were maintained in DMEM supplemented with 100 U of penicillin/ml, 100 mg of streptomycin/ml, 10% FCS, and 2 mM l-glutamate (cMEM). Cells were maintained at 37 °C in a 5% CO_2_ environment. MEF cells were prepared from C57BL/6 as previously described [89]. Vero-Cre cells were a gift from David Leib and were passaged in cMEM supplemented with 300 μg of hygromycin B/ml (Calbiochem, http://www.calbiochem.com).

### Generation of virus mutants.

The γHV68 genome cloned as a BAC was a kind gift of Ulrich Kozinowski [41]. For the generation of the recombinant γHV68-IκBαM.1 and .2 viruses, the mutant superrepressor form of IκBα that contains serine-to-alanine substitutions at amino acids 32 and 36 (IκBαM) driven by the CMV immediate-early promoter was inserted into the ORF27–ORF29b intergenic region of γHV68. The 1.8-kb AseI-MluI fragment of pIκBαM (Stratagene, http://www.stratagene.com) was cloned into the PmlI site (corresponding to γHV68 genomic position 46347) of plasmid JE110 [32] that contains the γHV68 genomic region between nucleotide positions 45237 and 48347. Insertion of the IκBαM expression construct into the PmlI site of pJE110 in the rightward and leftward orientation generated JE110-IκBαM.1 and JE110-IκBαM.2, respectively. To generate the targeting construct for use in recombination, the BglII-EcoRI fragment of pJE110-IκBαM was cloned into the suicide donor plasmid pGS284 to generate pGS284-IκBαM. Allelic exchange with WT γHV68-BAC in GS500 E. coli (RecA^+^) cells was performed as previously described [90], and the insertion of IκBαM to generate recombinant γHV68-BAC-IκBαM.1 and .2 was screened by colony PCR using primers ACGGGCTGAAGAAGGAGCGGCTACT and TGCCCAGGTAGCCATGGATAGAGG with *Taq* DNA polymerase (Promega, http://www.promega.com). For generation of the marker rescue virus, the BglII-EcoRI fragment of pJE110 was cloned into pGS284 to generate pGS284-27-29B. Allelic exchange of this targeting plasmid with γHV68-IκBαM.1-BAC was performed to generate γHV68-IκBαM.MR. Loss of IκBαM was verified by diagnostic PCR as described above.

Viral stocks were generated by Superfect (Qiagen, http://www.qiagen.com) transfection of BAC clones of γHV68-IκBαM.1 and .2 or γHV68-IκBαM.MR into Vero-Cre cells. Following the appearance of cytopathic effect, wells were harvested and used to infect Vero-Cre cells to generate high-titer viral stocks.

### Purification and analysis of virion DNA.

Virus was isolated from the supernatants of infected cells when monolayers exhibited greater than 50% cytopathic effect. After two rounds of 20-min room-temperature spins at 2,000 × *g,* clarified supernatants were spun at 15,000 × *g* for 2 h at 4 °C. The pellet was rinsed with PBS, resuspended in 50 mM Tris (pH 7.5), 10 mM MgCl_2_, and 25 U of DNase (Invitrogen, http://www.invitrogen.com), and incubated for 35 min at 37 °C before being layered over a 20% sucrose cushion and centrifuged at 110,000 × *g* for 1 h at room temperature. The virion pellet was resuspended in 3 ml of 10 mM Tris (pH 7.5), 1 mM EDTA and mixed with 3 ml of a 2× lysis buffer containing 2% Sarkosyl, 0.5% SDS, 40 mM Tris (pH 7.5), 200 mM NaCl, 20 mM EDTA, and 333 μg/ml proteinase K (GIBCO-BRL, http://www.gibco.com) and then incubated at 37 °C overnight. Samples were subjected to a phenol/chloroform and chloroform extraction, and DNA was precipitated with 3 M Na aAcetate and 2 volumes of 100% ethanol.

Southern analysis was performed with a HindIII restriction digest of the viral DNA and a ^32^P-labeled probe that detects ORF27 and the intergenic region between ORFs 27 and 29b (bp 45237 to 46420 of γHV68 WUMS) and with a BamHI restriction digest of the viral DNA and a ^32^P-labeled IκBαM probe (BamHI-XhoI fragment of pIκBαM).

### Mice, infections, and organ harvests.

Female C57BL/6J mice (catalog No. 000664; The Jackson Laboratory, http://www.jax.org) were housed at the Yerkes vivarium in accordance with federal and university guidelines. C57BL/6-Tg(BCL2)22Wehi/J (catalog No. 002319; The Jackson Laboratory) were maintained as heterozygous animals by breeding with C57BL6/6J mice under sterile breeding conditions at the Yerkes vivarium. Tg(BCL2) mice contain a transgene construct consisting of the human BCL2 cDNA driven by the Emu immunoglobulin heavy chain enhancer and SV40 promoter; BCL2 expression is thereby restricted to the B-cell lineage [60]. All protocols for animal studies were approved by the Institutional Animal Care and Use Committee of Emory University.

Mice between 8 and 12 wk of age were placed under isofluorane anesthesia prior to intranasal inoculation with 1,000 PFU or 1 × 10^5^ PFU of virus in 20 μl of cMEM or intraperitoneal inoculation of 1,000 PFU of virus in 0.5 ml of cMEM. Spleens were harvested into cMEM, homogenized, and filtered through a 100-μm-pore nylon cell strainer (Becton Dickinson, http://www.bd.com). Erythrocytes were removed with red blood cell lysis buffer (Sigma, http://www.sigmaaldrich.com). Except where indicated, pooled splenocytes from three to 15 mice were used in all experiments. Lungs were harvested intact and briefly incubated in HEPES-buffered saline solution with 1.3 mM EDTA supplemented with 2% fetal calf serum (HBSS^++^) prior to inflation with approximately 1 ml of 300 U/ml collagenase type I (MOP4405; Worthington Biochemical, http://www.worthington-biochem.com) prepared in the HBSS^++^ solution, minced, and then incubated for 1 h at 37 °C. Collagenase-disrupted lungs were homogenized, filtered through a 100-μm cell strainer, treated with blood cell lysis buffer, and resuspended in PBS supplemented with 0.5% or 2% FCS, depending on downstream application.

### Antiviral therapy.

Individual mice were weighed prior to the first injection and weekly thereafter. Cidofovir (Vistide; Gilead Sciences, http://www.gilead.com) was administered subcutaneously in the scruff of the neck at a dose of 25 mg/kg of body weight [47,48] every 3 d between 16 and 39 dpi with 1,000 PFU of the viruses as indicated in the legend to Figure 5.

### Plaque assay.

Plaque assays were performed as previously described [53], with minor modifications. NIH 3T12 cells were plated onto six-well pates 1 d prior to infection at 2 × 10^5^ cells per well. Organs were subjected to four rounds of mechanical disruption of 1 min each using 1.0-mm zirconia/silica beads (Biospec Products, http://www.biospec.com) in a Mini-Beadbeater-8 (Biospec Products). Serial 10-fold dilutions of organ homogenate were plated onto NIH 3T12 monolayers in a 200-μl volume. Infections were performed for 1 h at 37 °C with rocking every 15 min. Immediately after infection, plates were overlaid with 2% methylcellulose in cMEM. After 6 to 7 d, plates were stained with a neutral red overlay, and plaques were scored the next day. The limit of detection for this assay is 50 PFU per organ.

### Reporter assay.

NIH 3T12 cells (3 × 10^5^) were seeded onto a six-well dish 1 d prior to treatment. Cells were infected at an MOI of 1 or 10 in 200 μl for 1 h at 37 °C with rocking every 15 min and then 2 ml of fresh medium was added. At 6 h after the addition of innoculum, a total of 2 μg of DNA per well was transfected by the lipofection-based method (LipofectAMINE Plus; Invitrogen). pNF-κBLuc is an NF-κB–responsive reporter construct that expresses Photinus pyralis luciferase (Stratagene). pMEKK expresses an activator of NF-κB (Stratagene). pIκBαM expresses IκBα S32/36, a dominant inhibitor of NF-κB (Stratagene), and pBSIIKS^+^ (Stratagene) is an empty vector used to bring up DNA content to equivalent levels for each transfection. pEGFP^+^ (Stratagene) was used to monitor transfection efficiency by immunofluorescence microscopy (Nikon, http://www.nikon.com). Cell extracts were harvested 24 h posttransfection and assayed using the luciferase reporter assay system (Promega). Transfections were performed independently in triplicate at least two times.

### Nuclear extract preparation and electrophoretic mobility shift analysis.

Cells were harvested, washed once in PBS, and resuspended into ice-cold hypotonic lysis buffer (10 mM HEPES [pH 7.9], 10 mM KCl, 1.5 mM MgCl_2_, 0.1 mM EDTA, 1 mM dithitothreitol, 0.5 mM phenylmethylsulfonyl fluoride, protease inhibitor cocktail; Roche, http://www.roche.com) for 15 min prior to the addition of 1/20 volume of 10% Nonidet P-40. Nuclei were spun down, washed in hypotonic lysis buffer, and then resuspended in high salt buffer (25% glycerol, 20 mM HEPES [pH 7.9], 420 mM NaCl, 1.5 mM MgCl_2_, 0.2 mM EDTA, 0.5 mM dithitothreitol, 0.5 mM phenylmethylsulfonyl fluoride, protease inhibitor cocktail; Roche) with vigorous shaking at 4 °C. The supernatant collected after 10,000 × *g* centrifugation for 10 min at 4 °C was nuclear extract.

Nuclear extracts were assayed for NF-κB activation by electrophoretic mobility shift assay. Five micrograms of nuclear extract were incubated with a ^32^P-labeled oligonucleotide containing the NF-κB consensus site, AGTTGAGGGGACTTTCCCAGGC, in a binding reaction containing 2 mM HEPES (pH 7.9), 1 mM EDTA, 5 mM dithiothreitol, 0.05% Triton X-100, 5% glycerol, and 2 μg of poly d(I-C) (Roche) for 30 min at room temperature. Competition experiments were performed with 20- and 100-fold molar excess of unlabeled oligonucleotides containing the WT or mutated NF-κB consensus site (AGTTGAGG*C*GACTTTCCCAGGC). Nucleoprotein complexes were subjected to electrophoresis in 5% native polyacrylamide gels at 180 V, dried under vacuum, and analyzed by PhosphorImager analysis (Typhoon 9410; Amersham Biosciences, http://www.amersham.com).

### Antibodies for flow cytometry.

Cells resuspended in PBS supplemented with 2% FCS were stained for FACS using a combination of the following antibodies: phycoerythrin (PE)- or allophycocyanin-conjugated antibodies to CD19; fluorescein isothiocyanate (FITC)-conjugated antibodies to B220, IgD, CD69, PNA, Ki67, and the IgG1 isotype control. When necessary, rat anti-mouse CD16/CD32 (Fc block) was used to block Fc receptors prior to staining. All reagents were obtained from BD Biosciences (http://www.bdbiosciences.com) except the FITC-conjugated Ki67 (Novocastra Laboratories, http://www.ebiotrade.com/buyf/Novocastra/index.htm).

### Flow cytometry.

For FACS, cells were resuspended at 2 × 10^7^ cells/ml and incubated for 30 min on ice in PBS containing 1% FCS and Fc block. Cells were stained with PE-conjugated anti-CD19 at 5 μl per 1 × 10^7^ cells by incubation for 30 min on ice in the dark. Cells were washed twice with PBS containing 1% FCS and resuspended at 1 × 10^8^ cells/ml. Stained cell populations were acquired using a MoFlo fluorescence activated cell sorter (DAKO, http://www.dako.com), FACSAria (BD Biosciences), or FACSVantage (BD Biosciences). Sorted and unsorted cell populations were resuspended in cMEM supplemented with 10% dimethyl sulfoxide and stored at −80 °C for limiting-dilution PCR analyses or in cMEM at 4 °C for limiting-dilution ex vivo reactivation analyses as described below.

For flow cytometry analysis, cells were resuspended at 1 × 10^6^ cells/ml in PBS containing 2% FCS for 20 min on ice in the dark and stained with 1:100 dilution of allophycocyanin-conjugated anti-CD19 and 1:100 to 1:200 dilution of FITC-conjugated antibodies. For intracellular staining for the detection of the Ki67 nuclear antigen, cells were surface stained prior to fixation in 2% paraformaldehyde and permeabilization with 0.1% Triton X-100 in PBS, followed by incubation with 1:100 dilution of the FITC-conjugated anti-Ki67 antibody or the isotype control. Data were collected on an FACSCaliber (BD Biosciences) and analyzed using FlowJo software (TreeStar, http://www.treestar.com).

### Magnetic cell separation.

Murine B cells were isolated by depletion of non–B cells using the B Cell Isolation Kit (Miltenyi Biotec, http://www.miltenyibiotec.com) per the manufacturer's recommendations. Briefly, cells were resuspended at 2 × 10^8^ cells/ml in 1× PBS containing 0.5% FCS followed by staining with Fc block (0.125 μg/10^6^ cells) on ice for 15 min. Cells were labeled with biotin-antibody cocktail (biotin-conjugated antibodies against CD43, CD4, and Ter-119), 10 μl per 1 × 10^7^ cells for 15 min on ice followed by staining with anti-biotin microbeads, 20 μl per 1 × 10^7^ cells for 15 min on ice. Cells were washed twice with PBS containing 0.5% FCS and subjected to magnetic separation using the autoMACS (Miltenyi Biotec). Following separation, stained cell populations were analyzed by flow cytometry as described above.

Lung B cells were isolated by positive selection of B cells using a PE-conjugated antibody against CD19 and the PE isolation kit per the manufacturer's recommendations (Miltenyi Biotec). Briefly, cells were resuspended at 2 × 10^8^ cells/ml in 1× PBS containing 0.5% FCS followed by staining with Fc block (0.125 μg/10^6^ cells) on ice for 15 min. Cells were labeled with CD19 conjugated to PE, 5 μl per 1 × 10^7^ cells for 30 min on ice in the dark, spun out, and resuspended in 80 μl of PBS containing 0.5% FCS per 1 × 10^7^ cells followed by staining with anti-PE microbeads, 20 μl per 1 × 10^7^ cells, for 15 min on ice in the dark. Cells were washed twice with PBS containing 0.5% FCS, filtered through 40-μm cell strainers, and subjected to magnetic separation using the autoMACS (Miltenyi Biotec).

### Limiting-dilution ex vivo reactivation analyses.

Limiting-dilution analysis to determine the frequency of cells containing virus capable of reactivating from latency was performed as previously described [22,26]. Briefly, bulk splenocytes or sorted cell populations were resuspended in cMEM and plated in serial 2-fold dilutions (starting with 10^5^ cells) onto MEF monolayers in 96-well tissue culture plates. Twelve dilutions were plated per sample, and 24 wells were plated per dilution. Wells were scored for cytopathic effect at 21 to 28 d postplating. To detect preformed infectious virus, parallel samples of mechanically disrupted cells were plated onto MEF monolayers. This process kills more than 99% of live cells, which allows preformed infectious virus to be discerned from virus reactivating from latently infected cells [22,26]. Unless indicated otherwise, significant levels of preformed virus were not detected in these assays.

### Limiting-dilution nested-PCR detection of γHV68 genome-positive cells.

Limiting-dilution analysis to determine the frequency of cells harboring the viral genome was performed using a single-copy-sensitive nested PCR assay as previously described [24,26]. Briefly, frozen samples were thawed, counted, resuspended in isotonic buffer, and plated in serial 3-fold dilutions in a background of 10^4^ uninfected NIH 3T12 cells in 96-well plates (Eppendorf Scientific, http://www.eppendorf.com). Plates were covered with PCR foil (Eppendorf Scientific), and cells were lysed with proteinase K for 6 h at 56 °C. Then, 10 μl of round 1 PCR mix was added to each well by foil puncture. Following first-round PCR, 10 μl of round 2 PCR mix was added to each well by foil puncture and samples were subjected to round 2 PCR. All cell lysis and PCR were performed on a PrimusHT thermal cycler (MWG Biotech, http://www.mwg-biotech.com). Products were resolved by ethidium bromide staining on 2% agarose gels. Twelve PCRs were performed for each sample dilution, and a total of six dilutions were performed per sample. Every PCR plate contained control reactions (uninfected cells and ten copies, one copy, 0.1 copy of plasmid DNA in a background of 10^4^ cells). All of the assays demonstrated approximately single-copy sensitivity with no false positives.

### Recovery of γHV68-IκBαM viruses from splenocytes.

γHV68-IκBαM.R1 and .R2 viruses were recovered from splenocytes harvested 16 dpi. Cytopathic effect was observed in wells at 14 d postplating. Wells that exhibited viral cytopathic effect and contained the fewest number of input splenocytes were harvested and used to infect NIH 3T12 fibroblasts to generate high-titer viral stocks and viral DNA.

### Statistical analyses.

All data were analyzed by using GraphPad Prism software (GraphPad Software, http://www.graphpad.com). Titer data were statistically analyzed with the Mann-Whitney nonparametric two-tailed *t*-test. Based on the Poisson distribution, the frequencies of reactivation and viral genome–positive cells were obtained from the nonlinear regression fit of the data where the regression line intersected 63.2%. The frequencies of reactivation and genome-positive cells were statistically analyzed by unpaired two-tailed *t*-test of the log 63.2% effective concentration.

## Supporting Information

### Accession Number

The GenBank (http://www.ncbi.nlm.nih.gov/Genbank) sequence for γHV68 WUMS is U97553.

## Abbreviations

γHV68: gammaherpesvirus 68
BAFF: B-cell–activating factor
dpi: days postinfection
CMV: cytomegalovirus
COX-2: cyclooxygenase 2
EBV: Epstein-Barr virus
FACS: fluorescence-activated cell sorting
FITC: fluorescein isothiocyanate
HCMV: human cytomegalovirus
hpi: hours postinfection
IKK: IκB kinase
IL: interleukin
KSHV: Kaposi sarcoma–associated herpesvirus
LMP: latent membrane protein
MEF: mouse embryonic fibroblast
MIEP: major immediate-early promoter
MOI: multiplicity of infection
NF-κB: nuclear factor-κB
ORF: open reading frame
PE: phycoerythrin
PEC: peritoneal exudate cell
PFU: plaque-forming unit
RANTES: regulated on activation, normal T-cell expressed and secreted
RTA: replication and transcription activator
TNF: tumor necrosis factor
vGPCR: viral G protein–coupled receptor
WT: wild-type
